# Statistical issues related to dietary intake as the response variable in intervention trials

**DOI:** 10.1002/sim.7011

**Published:** 2016-06-20

**Authors:** Ruth H. Keogh, Raymond J. Carroll, Janet A. Tooze, Sharon I. Kirkpatrick, Laurence S. Freedman

**Affiliations:** ^1^ Department of Medical StatisticsLondon School of Hygiene and Tropical MedicineLondonU.K.; ^2^ Department of StatisticsTexas A&M University3143 TAMUCollege StationTX77843‐3143U.S.A.; ^3^ School of Mathematical and Physical SciencesUniversity of Technology SydneyBroadwayNew South Wales2007Australia; ^4^ Department of Biostatistical SciencesWake Forest School of MedicineWinston‐SalemNCU.S.A.; ^5^ School of Public Health and Health SystemsUniversity of WaterlooWaterlooOntarioCanada; ^6^ Information Management Services, Inc.RockvilleMDU.S.A.; ^7^ Biostatistics UnitGertner Institute for Epidemiology and Health Policy Research, Sheba Medical CenterRamat GanIsrael

**Keywords:** biomarker, differential error, intervention trial, measurement error, self‐report, sodium intake

## Abstract

The focus of this paper is dietary intervention trials. We explore the statistical issues involved when the response variable, intake of a food or nutrient, is based on self‐report data that are subject to inherent measurement error. There has been little work on handling error in this context. A particular feature of self‐reported dietary intake data is that the error may be differential by intervention group. Measurement error methods require information on the nature of the errors in the self‐report data. We assume that there is a calibration sub‐study in which unbiased biomarker data are available. We outline methods for handling measurement error in this setting and use theory and simulations to investigate how self‐report and biomarker data may be combined to estimate the intervention effect. Methods are illustrated using data from the Trial of Nonpharmacologic Intervention in the Elderly, in which the intervention was a sodium‐lowering diet and the response was sodium intake. Simulations are used to investigate the methods under differential error, differing reliability of self‐reports relative to biomarkers and different proportions of individuals in the calibration sub‐study. When the reliability of self‐report measurements is comparable with that of the biomarker, it is advantageous to use the self‐report data in addition to the biomarker to estimate the intervention effect. If, however, the reliability of the self‐report data is low compared with that in the biomarker, then, there is little to be gained by using the self‐report data. Our findings have important implications for the design of dietary intervention trials. © 2016 The Authors. *Statistics in Medicine* published by John Wiley & Sons Ltd.

## 1 Introduction

Our health is undeniably linked with what we eat, and there is a long history of studies that aimed to discover the effects of diet on health, starting with what is probably the earliest recorded intervention study 1, and including the famous scurvy experiment of James Lind 2. Nowadays, based on known links between diet and health, such as salt intake with blood pressure 3, saturated fat intake with serum low density lipoprotein cholesterol levels 4, and energy balance with obesity 5, investigators are studying also how to get individuals to eat a healthier diet. In these studies, the focus is often on the intermediate aim of achieving dietary change 6, 7, 8. The appropriate response variables in such studies are the intakes of the nutrients or foods that are the targets for change. In other studies, the main focus is on the effects of the intervention on a health outcome that may, at least in part, be mediated through dietary change. In this case, the dietary response variable is a secondary outcome. Sometimes, dietary outcomes can be measured objectively using a reliable biomarker, such as 24‐h urinary sodium 9, but sometimes, no such objective measure exists or is prohibitively expensive. This paper explores the statistical issues involved when the main response variable of interest is to be based instead on self‐reported dietary intake data.

A primary concern when considering the use of a reported dietary intake is the measurement error inherent in self‐report data. Measurement error is an important barrier to progress in many areas of scientific endeavor and has been particularly difficult in the field of nutritional epidemiology 10. Depending on the context, measurement error will at best reduce the precision with which a study can estimate the effect of interest and at worst could produce spurious results or fail to identify true effects. To counter this problem, various statistical methods for dealing with measurement error have been developed, leading to a growing body of literature on this topic. Although the statistical literature on measurement error is vast, surprisingly, the focus has been greatly concentrated on problems where explanatory variables in regression models are measured with error 11, 12, 13, and rather little has been written on the problem where the response (or outcome) variable is measured with error 14. For example, in the book of Carroll *et al*. 11, there are 15 chapters, and only the last deals with error in the response variable.

Implementation of methods for dealing with measurement error requires information on the nature of the measurement errors in the self‐report data. This requires sub‐studies that have been conducted alongside the main study to assess the magnitude and nature of error in the measurements of the key variables, by comparing the main self‐report instrument with more accurate indicators, such as biomarkers, that can be assumed to be unbiased measures of dietary intake 15. The more accurate measures, which we will here refer to as biomarker measurements, may be considered to provide a gold‐standard measure of intake of the dietary components of interest but are usually expensive and cannot necessarily be obtained for all participants in the study. Therefore, a compromise design would be one that included self‐report data for all the participants, and biomarker data on a subset. The biomarker portion of the study is often called a calibration sub‐study, because it provides a means of ‘calibrating’ the self‐report to the biomarker.

In our application, dietary intake is the outcome measure following the intervention, and we are concerned with handling measurement errors in this context. We will draw on the work already performed on this problem, particularly by Buonaccorsi 14, and will develop it in new directions. In particular, we will discuss the problem of differential measurement error, where the magnitude of the bias differs between the intervention and control groups. This can be a critical concern for self‐reported intake in dietary intervention studies because those exposed to the intervention may be more prone to altering the reporting of their intake, which is thought to occur frequently in such studies (e.g., 16). We will address the following questions, using theory, a real example, and simulations based on data from the real example:

- What methods can be used to take advantage of the combined data from a calibration sub‐study where biomarkers are available and data on self‐reports from the main study to estimate the intervention effect?
- In what circumstances are the self‐report data helpful in addition to the biomarker data from the calibration sub‐study for estimating the intervention effect?

The paper is organized as follows. In Section 2, we outline the situation in statistical terms and introduce the notation. Methods for estimation of the intervention effect using the combined data from the calibration sub‐study and the main study are outlined in Section 3. In Section 4, the methods are illustrated using data from the Trial of Nonpharmacologic Intervention in the Elderly (TONE), in which the intervention was a sodium‐lowering diet and the outcome of interest was sodium intake 17. In this study, the dietary outcome was a secondary outcome. In Section 5, detailed simulation studies based on data from TONE are used to assess the performance of the methods across a range of scenarios, and in Section 6, we conclude with a discussion and recommendations.

## 2 Models for error in a dietary outcome

We state the problem in statistical terms as follows. We have a set of individuals who have been allocated by randomization to either an intervention or a control group. The intervention is designed to change their intake of a particular dietary component (nutrient or food). At a chosen point of time, usually the end of the intervention period, the true intake (or change in intake) of individual *j* (*j* = 1, …, *N*_*i*_) in group *i* (*i* = 1 for control and 2 for intervention) is denoted by *T*_*ij*_ .

A simple model for *T*_*ij*_ is:

(1) $$T_{ij}=\mu_{Ti}+\epsilon_{T_{ij}}\text{,}$$

where the $\epsilon_{T_{ij}}$ have expectations zero for each group *i* and variances $\sigma_{T_{i}}^{2}$ and *μ*_*Ti*_ (*i* = 1, 2) is the expected intake (or change in intake) under control or intervention, respectively. The main aim of the study is to estimate the intervention effect *μ*_*T*2_ − *μ*_*T*1_. The focus is assumed to be on the absolute intake of the nutrient or food at a given point in time, although the methods that we describe could also be applied to a situation in which the focus is on a change in dietary intake. This would require self‐report data in the main study and biomarker data in the calibration sub‐study to be available at both time points from which the change is calculated.

Unfortunately, we cannot measure *T*_*ij*_. Instead, we have the following data. First, we have a measure of intake at the end of the intervention period obtained from the study participants by self‐report, for example, using a questionnaire or 24‐h recall. We denote these data by *Q*_*ij*_. These measurements are related to true intake, but due to being self‐reported, they are not considered to provide an unbiased measure of it 10. We write the statistical model for the self‐report as

(2) $$Q_{ij}=\alpha_{0i}+\alpha_{1i}T_{ij}+{\epsilon_{Q}}_{ij}\text{,}$$

where the *ϵ*_*Qij*_ are independent random errors with means zero and variances $\sigma_{Q_{i}}^{2}\;\left(i=1,2\right)$. The parameters *α*_0*i*_ and *α*_1*i*_ are the intercepts and slopes, respectively, in a linear regression of *Q* on *T* for each treatment group *i* (*i* = 1, 2). The measurement error is in general non‐classical; only if *α*_0*i*_ = 0 and *α*_1*i*_ = 1 is the measurement error classical and the self‐report unbiased. Model (2), in its generality, describes the situation of differential error between the intervention and control groups, because the *α*‐quantities differ between the two groups. Only when *α*_01_ = *α*_02_ and *α*_11_ = *α*_12_ is the error non‐differential. We use the term ‘differential error’, although, traditionally in this area of statistics, differential error refers to error in an explanatory variable differing according to the outcome variable. In our case, the outcome itself (*T*) is measured with error, and the differential error occurs when the error in the outcome differs according to the explanatory variable, the treatment group variable.

Second, we have a series of *K*_*ij*_ biomarker measurements of intake in each treatment group in a subset of *n*_*i*_ individuals. Without loss of generality, individuals *j* = 1, …, *n*_*i*_ are assumed to be in the sub‐study in treatment group *i* and individuals *n*_*i*_ + 1, …, *N*_*i*_ outside the sub‐study. We denote these biomarker data *M*_*ijk*_ (*i* = 1, 2; *j* = 1, …, *n*_*i*_; *k* = 1, …, *K*_*ij*_). It is assumed that these measures are made close enough in time to the time point of interest for the dietary outcome that they measure the intake of interest. These biomarker measurements are unbiased measures of *T*_*ij*_ but have some random error. We write their statistical model as

(3) $$M_{ijk}=T_{ij}+{\epsilon_{M}}_{ijk}\text{,}$$

where *ϵ*_*Mijk*_ are independent random errors with means zero and variances $\sigma_{Mi}^{2}$ (*i* = 1, 2).

We define the reliability of the self‐report to be the correlation between repeat reports by the same person; from (2), this equals *α*_1*i*_^2^var(*T*_*ij*_)/var(*Q*_*ij*_). Similarly, the reliability of the biomarker is var(*T*_*ij*_)/var(*M*_*ijk*_). The reliabilities of the self‐report and the biomarker are clearly reduced as the error variances, $\sigma_{\text{Qi}}^{2}$ and $\sigma_{Mi}^{2}$ respectively, increase.

For convenience of developing the methods, we will assume that the error terms in models (1), (2), (3) are independent of each other and normally distributed. In practice, with dietary data, this assumption can often be satisfied by using a logarithmic or power transformation of the data. For the methods described in the next section to be applied, at least some individuals in the calibration sub‐study must have at least two biomarker measurements, that is, *K_ij_* > 1 for some values of (*i*,*j*).

We let μ_*Qi*_ denote the expected self‐report measurement in treatment group *i* (*i* = 1, 2), and μ_*Mi*_ denote the expected biomarker measurement. A consequence of model (2) is that the expected difference between self‐report measurements in the two groups, $\mu_{Q_{2}}-\mu_{Q_{1}}$, is not equal to the true intervention effect, *μ*_*T*2_ − *μ*_*T*1_. In fact, we have $\mu_{Q_{2}}-\mu_{Q_{1}}=\left(\alpha_{02}-\alpha_{01}\right)+\left(\alpha_{12}\mu_{T2}-\alpha_{11}\mu_{T1}\right)$. It follows that the simple difference between means of the self‐report in the two groups, ${\bar{Q}}_{2}-{\bar{Q}}_{1}$, does not provide an unbiased estimate of the true intervention effect. Therefore, to use the self‐report data in estimating the intervention effect, one needs to employ non‐standard methods. A consequence of the classical error model for the biomarkers in (3) is that $\mu_{M_{2}}-\mu_{M_{1}}=\mu_{T2}-\mu_{T1}$. It follows that a difference in means of the biomarker measurements in the two treatment groups does provide an unbiased estimate of the intervention effect. However, biomarker measurements are available only in the calibration sub‐study, which may be a small proportion of the main study. This raises the question of how data on biomarkers from a calibration sub‐study can be combined with data from self‐reports to efficiently estimate the intervention effect.

## 3 Methods for estimating the intervention effect

In this section, we outline methods for estimation of the intervention effect using data from self‐reports in the main study and data from biomarkers in a calibration sub‐study. We focus on two approaches: the first using method of moment estimation and the second using maximum likelihood estimation. Although the maximum likelihood approach may be more efficient than the methods of moments approach, the latter enables us to show clearly and intuitively the relative contribution of the biomarkers and self‐reports to the intervention effect estimate. Also, in practice, the efficiency gain from using maximum likelihood may be rather small.

### 3.1 The Buonaccorsi approach using the method of moments

With models (2) and (3) as the background, Carroll *et al*. [11: Chapter 15] describe an approach to estimation based on a series of papers by Buonaccorsi and colleagues 14, 18, 19. We suppose for simplicity here that the number of repeats of the biomarker, *K_ij_*, is the same (*K*) for all individuals in the sub‐study and is greater than 1. The main ideas of the approach, applied to our setting, are

- The statistic ${\hat{\theta }}_{\left(i\right)}={\hat{\mu }}_{M_{2}}-{{\hat{\mu }}_{M}}_{1}$ is an unbiased estimator of the intervention effect *μ*_*T*2_ − *μ*_*T*1_, where ${\hat{\mu }}_{M_{i}}={\bar{M}}_{i}=\frac{1}{n_{i}}\sum_{j=1}^{n_{i}}\sum_{k=1}^{K}M_{ijk}/K$.
- The statistic ${\hat{\theta }}_{\left(ii\right)}=\frac{{{\hat{\mu }}_{Q}}_{2}-{\hat{\alpha }}_{02}}{{\hat{\alpha }}_{12}}-\frac{{{\hat{\mu }}_{Q}}_{1}-{\hat{\alpha }}_{01}}{{\hat{\alpha }}_{11}}$ is a consistent estimator of the intervention effect *μ*_*T*2_ − *μ*_*T*1_, where ${{\hat{\mu }}_{Q}}_{i}={\bar{Q}}_{i}=\frac{1}{N_{i}}\sum_{j=1}^{N_{i}}Q_{ij}$.

The hats on the *α*‐quantities in (ii) denote estimates that are derived from an errors‐in‐variables analysis of the regression of Q on M in the subsets of individuals who have biomarker measurements. We discuss their estimation in the succeeding discussion.

The Buonaccorsi approach is to combine the estimates in (i) and (ii) by taking their weighted average, using weights that take into account the variances and covariance of the two estimates so as to minimize the variance of the combination.

We investigate what the contribution of statistic ${\hat{\theta }}_{\left(ii\right)}$ to the overall estimator is under different assumptions about the form of the error in the self‐report. Two sets of assumptions are considered:

*Differential error*: This is the most general case in which there are no restrictions on the *α*‐quantities in model (2).
*Non‐differential error*: This is the special case in which the slope and intercept parameters in model (2) are the same in the two treatment groups, that is, *α*_01_ = *α*_02_ = *α*_0_, say, and *α*_11_ = *α*_12_ = *α*_1_, say.

For simplicity and to emphasize the main points, in the methods described in the succeeding discussion, we assume that there are two biomarker measurements (*K* = 2) for each individual in the calibration sub‐study.

#### 3.1.1 Differential error

If the error in the self‐report is differential, method of moments estimators for *α*_1*i*_ and *α*_0*i*_ (*i* = 1, 2) are

(4) $${\hat{\alpha }}_{1i}=\frac{cov\left(Q_{ij},{\bar{M}}_{ij}\right)}{cov\left(M_{ij1},M_{ij2}\right)}\text{,}$$

(5) $${\hat{\alpha }}_{0i}={\bar{Q}}_{i}^{\left(s\right)}-{\hat{\alpha }}_{1i}{\bar{M}}_{i}\text{,}$$

where the covariances in (4) are obtained among those in treatment *i* group and in the sub‐study, ${\bar{Q}}_{i}^{\left(s\right)}$ (*i* = 1, 2) denotes the mean of the self‐reports among individuals in the sub‐study and in treatment group *i* ${\bar{Q}}_{i}^{\left(s\right)}=\frac{1}{n_{i}}\sum_{j=1}^{n_{i}}Q_{ij}$, and ${\bar{M}}_{ij}=\sum_{k=1}^{K}M_{ijk}/K$.

Substituting these estimates into the expression for ${\hat{\theta }}_{\left(ii\right)}$, we obtain

(6) $${\hat{\theta }}_{\left(ii\right)}=\frac{{{\hat{\mu }}_{Q}}_{2}-{\hat{\alpha }}_{02}}{{\hat{\alpha }}_{12}}-\frac{{{\hat{\mu }}_{Q}}_{1}-{\hat{\alpha }}_{01}}{{\hat{\alpha }}_{11}}=\frac{{\bar{Q}}_{2}-{\bar{Q}}_{2}^{\left(s\right)}}{{\hat{\alpha }}_{12}}-\frac{{\bar{Q}}_{1}-{\bar{Q}}_{1}^{\left(s\right)}}{{\hat{\alpha }}_{11}}+{\bar{M}}_{2}-{\bar{M}}_{1}$$

Because, usually, the patients who participate in the sub‐study are chosen to be representative of those in the main study, ideally selected at random, one expects the quantities ${\bar{Q}}_{i}-{\bar{Q}}_{i}^{\left(s\right)}\;\left(i=1,2\right)$ to approach zero as the sample size increases. Hence, for very large sample sizes, the statistic in (6) using self‐reports will be very close to the statistic in ${\hat{\theta }}_{\left(i\right)}={\bar{M}}_{2}-{\bar{M}}_{1}$. In that case, statistic ${\hat{\theta }}_{\left(ii\right)}$ will add little information to that in ${\hat{\theta }}_{\left(i\right)}$. Consequently, nearly all the information about the intervention effect will come from the biomarker measurements, and very little from the self‐reported intakes. The question arises, however, whether this is true for the sample sizes and levels of measurement error that are typical in dietary intervention studies. The overall mean of the self‐report ${\bar{Q}}_{i}$ and the mean in the sub‐study ${\bar{Q}}_{i}^{\left(s\right)}$ may be quite different for finite overall sample size when the proportion of individuals in the sub‐study is small.

#### 3.1.2 Non‐differential error

Under the assumption of non‐differential error in the self‐reports, the method of moments estimators for *α*_0_ and *α*_1_ are

(7) $${\hat{\alpha }}_{1}=\frac{cov\left(Q_{ij},{\bar{M}}_{ij}\right)}{cov\left(M_{ij1},M_{ij2}\right)}\text{,}$$

(8) $${\hat{\alpha }}_{0}={\bar{Q}}^{\left(s\right)}-{\hat{\alpha }}_{1}\bar{M}\text{,}$$

where ${\bar{Q}}^{\left(s\right)}=\frac{1}{n}\sum_{i=1}^{2}\sum_{j=1}^{n_{i}}Q_{ij}$, $\bar{M}=\frac{1}{n\;}\sum_{i=1}^{2}\sum_{j=1}^{n_{i}}\sum_{k=1}^{K}M_{ijk}/K$ and $n=n_{1}+n_{2}$ and where the covariances in (7) are obtained using the combined data from the two treatment groups *i* = 1, 2 from the sub‐study. In this case, therefore, the statistic based on the self‐reports, ${\hat{\theta }}_{\left(ii\right)}=\frac{{\bar{Q}}_{2}-{\bar{Q}}_{1}}{{\hat{\alpha }}_{1}}$, provides a consistent estimator of the intervention effect *μ*_*T*2_ − *μ*_*T*1_. Thus, when the parameters *α*_0_, *α*_1_ are common to both treatment groups, ${\hat{\theta }}_{\left(ii\right)}$ does not reduce to ${\hat{\theta }}_{\left(i\right)}$ for large sample size, and hence, we expect that the combined estimate of the intervention effect using ${\hat{\theta }}_{\left(i\right)}$ and ${\hat{\theta }}_{\left(ii\right)}$, that is, an estimate based on both biomarkers and self‐reports, will be more precise than that estimated using the biomarkers only, that is, ${\hat{\theta }}_{\left(i\right)}$.

#### 3.1.3 Combination of estimates

To obtain the combined intervention effect estimate, we take an inverse‐variance‐weighted average of ${\hat{\theta }}_{\left(i\right)}$ and ${\hat{\theta }}_{\left(ii\right)}$. The inverse‐variance‐weighted combined estimate has weights that are designed to minimize the variance of the estimator. Because the estimate based on biomarkers only is equivalent to using weights of 1 and 0 for the two components, respectively, it can be thought of as a combined estimate with non‐optimal weights that will have variance larger than the inverse‐variance‐weighted combined estimate. Because ${\hat{\theta }}_{\left(i\right)}$ and ${\hat{\theta }}_{\left(ii\right)}$ are functions of other parameter estimates, we use an estimating equations approach and linear approximations to obtain the variances of the two estimates and their covariance. Details of this approach are given in the Appendix S1, and example R code for implementation is also provided. From equation (A2), we see that in the combined estimate, the biomarkers estimate ${\hat{\theta }}_{\left(i\right)}$ receives a larger weight than the self‐report estimate ${\hat{\theta }}_{\left(ii\right)}$ whenever the variance of ${\hat{\theta }}_{\left(ii\right)}$ is greater than the variance of ${\hat{\theta }}_{\left(i\right)}$ and that the relative contribution of ${\hat{\theta }}_{\left(ii\right)}$ increases as $var\left({\hat{\theta }}_{\left(ii\right)}\right)/var\left({\hat{\theta }}_{\left(i\right)}\right)$ decreases. Under both differential and non‐differential error, smaller error variances in the self‐report ( $\sigma_{Q_{i}}^{2}$) will result in a smaller variance for ${\hat{\theta }}_{\left(ii\right)}$, $var\left({\hat{\theta }}_{\left(ii\right)}\right)$. Hence, we expect the self‐report to make a greater contribution to the combined estimate, via ${\hat{\theta }}_{\left(ii\right)}$, when the error variances are small. This is investigated in the simulation study. A method of moments estimate for the biomarkers error variance is ${\hat{\sigma }}_{Mi}^{2}=var\left(M_{ijk}\right)-cov\left(M_{ij1},M_{ij2}\right)$ in the case of differential error. A methods of moments estimate for the self‐report error variance is ${\hat{\sigma }}_{\text{Qi}}^{2}=var\left(Q_{ij}\right)-{\hat{\alpha }}_{1i}^{2}\;cov\left(M_{ij1},M_{ij2}\right)$ in the case of differential error, where ${\hat{\alpha }}_{1i}$ is obtained from the solution to the estimating equations given in Appendix S1. In the case of non‐differential error, ${\hat{\sigma }}_{M}^{2}$ and ${\hat{\sigma }}_{Q}^{2}$ are obtained using the same expressions, but the variances and covariances are obtained using both treatment groups combined, and ${\hat{\alpha }}_{1i}$ is replaced by ${\hat{\alpha }}_{1}$.

### 3.2 The maximum likelihood approach

The intervention effect can alternatively be estimated by maximum likelihood. Unlike the Buonaccorsi approach, this requires assumptions about the joint distribution of the biomarker and self‐report measurements. In the most general case of differential error, we suppose that *M*_1*j*1_, *M*_1*j*2_, *M*_2*j*1_, *M*_2*j*2_, *Q*_1*j*_, *Q*_2*j*_ are generated from a multivariate normal distribution with mean vector

(9) $${\left(\mu_{T1},\mu_{T1},\mu_{T2},\mu_{T2},\alpha_{01}+\alpha_{11}\mu_{T1},\alpha_{02}+\alpha_{12}\mu_{T2}\right)}^{T}$$

and covariance matrix

(10) $$\left(\begin{array}{l} \sigma_{T1}^{2}+\sigma_{M1}^{2}\;\sigma_{T1}^{2}\;0\;0\;a_{11}\sigma_{T1}^{2}\;0\\ \;\sigma_{T1}^{2}\;\sigma_{T1}^{2}+\sigma_{M1}^{2}\;0\;0\;a_{11}\sigma_{T1}^{2}\;0\\ \;0\;0\;\sigma_{T2}^{2}+\sigma_{M2}^{2}\;\sigma_{M2}^{2}\;0\;a_{12}\sigma_{T2}^{2}\\ \;0\;0\;\sigma_{M2}^{2}\;\sigma_{T2}^{2}+\sigma_{M2}^{2}\;0\;a_{12}\sigma_{T2}^{2}\;\\ \;a_{11}\sigma_{T1}^{2}\;a_{11}\sigma_{T1}^{2}\;0\;0\;a_{11}\sigma_{T1}^{2}+\sigma_{Q1}^{2}\;0\\ \;0\;0\;a_{12}\sigma_{T2}^{2}\;a_{12}\sigma_{T2}^{2}\;0\;a_{12}^{2}\sigma_{T2}^{2}+\sigma_{Q2}^{2} \end{array}\right)\text{.}$$

If we assume non‐differential error, then the mean vector and covariance matrix are altered so that *α*_01_ = *α*_02_ = *α*_0_ and *α*_11_ = *α*_12_ = *α*_1_. Under either set of assumptions (differential or non‐differential error in the self‐report measurements), it may be reasonable to assume that the variability in true intake is the same across the two treatment groups, $\sigma_{Ti}^{2}=\sigma_{T}^{2}\;\left(i=1,2\right)$, or that the variability of the errors in the self‐reports and biomarkers are the same in the two groups, $\sigma_{\text{Qi}}^{2}=\sigma_{Q}^{2},\;\sigma_{Mi}^{2}=\sigma_{M}^{2}\;\left(i=1,2\right)$. Such assumptions can be tested using likelihood ratio tests. Using maximum likelihood, the expected outcomes in the two groups, *μ*_*T*1_ and *μ*_*T*2_, are estimated directly alongside the other parameters, and the intervention effect follows by taking their difference.

In practice, the parameters are estimated by maximizing the full likelihood for the data. The full likelihood is formed from the product of the likelihood for the data in the sub‐study (*M*_*ij*1_, *M*_*ij*2_, *Q*_*ij*_; *j* = 1, …, *n*_*i*_, *i* = 1, 2) and that for the data outside the sub‐study (*Q*_*ij*_; *j* = *n*_*i*_ + 1, …, *N*_*i*_, *i* = 1, 2). The variance–covariance matrix for the maximum likelihood estimates (MLEs) can be estimated in the standard way using the inverse of the observed information matrix.

We note that in our setting of linear mixed models, all calculations for the MLEs are based only on the first and second moments of the observed data, so the MLEs are in fact methods of moment estimators. As such, the MLEs make no distributional assumptions and are valid even if the data do not follow a multivariate normal distribution. However, in this case, the standard variance estimates are not valid. Under departures from multivariable normality the variance–covariance matrix for the MLEs could be obtained by bootstrapping or by using sandwich estimates. The sandwich estimates approach is described in detail in Appendix S2.

## 4 Example: Trial of Nonpharmacologic Intervention in the Elderly (TONE)

The TONE study was a randomized controlled trial designed to investigate whether weight loss or reduction in sodium intake, or both, result in satisfactory blood pressure control in individuals taking antihypertensive medication after removal of the antihypertensive medication. Full details of the trial are given by Appel *et al.* 17. The primary endpoints were a blood pressure of 150/90 mmHg or higher, resumption of antihypertensive drug therapy, or the occurrence of a blood pressure‐related clinical complication during 2–3 years of follow‐up. Study participants were randomized to one of four intervention arms: sodium‐lowering diet, weight loss regime, sodium‐lowering diet plus weight loss regime, or no intervention. Individuals who were obese were randomized to one of the four intervention groups, while non‐obese individuals were randomized only to a sodium‐lowering diet or to usual care.

In this illustration, we combine the obese and non‐obese groups and focus on two intervention groups: *Group 1*: usual sodium diet (with or without weight loss regime); *Group 2*: sodium‐lowering diet (with or without weight loss regime). The intervention effect is defined as the difference between the expected sodium intake in the sodium‐lowering diet group and the expected sodium intake in the usual sodium diet group.

Self‐reported measures of nutrient intake were obtained using 24‐h recalls at several time points over the course of follow‐up. Urinary biomarker measures were also available at a subset of follow‐up times. We focus on the 24‐h recall measure of sodium intake at 9 months of follow‐up, *Q_ij_*. The first biomarker measure of the outcome, *M*_*ij*1_, was urinary sodium at 9 months. We use a biomarker measure made at 18 months of follow‐up as the repeated measure, *M*_*ij*2_.

The TONE study involved a total of 975 randomized individuals. Of these, 850 had a 24‐h measurement of sodium intake at the 9 month time point, 867 had the concurrent urinary sodium measure, and 804 had the repeated urinary sodium measure. We restrict our analyses to 751 individuals who had all three measurements observed; of these, 371 were in treatment Group 1 (usual sodium diet), and 380 were in treatment Group 2 (sodium‐lowering diet). Restricting our illustration to this subset of 751 individuals enables us to compare error‐corrected estimates of the treatment effect with estimates of the intervention effect using only biomarkers, which we consider to be the gold‐standard method. To assess error‐corrected estimates, we will set some of the biomarker measurements to be missing in varying proportions of the sample.

The self‐reports and the biomarker measurements had skewed distributions within treatment groups, so we transformed all measurements using a power of 0.3, as suggested by using a Box–Cox analysis 20. It is known that urinary sodium underestimates dietary intake of sodium and Holbrook *et al* 21 found that the mean percentage of dietary sodium excreted in the urine was 86%. To account for this in our example, we divided all urinary sodium measurements by 0.86. Thus, we assumed models (1), (2) and (3) for true intake, self‐reports and biomarkers respectively (with *M*_*ijk*_ replaced by *M*_*ijk*_/0.86 in (3)), for *i* = 1, 2; *j* = 1, …, *N*_*i*_; *k* = 1, 2.

We estimated the parameters of models (1), (2), (3) by maximum likelihood with no restrictions on the parameters in the model for the self‐report, which is under differential error. Standard errors were estimated in the standard way using the Fisher information matrix. The complete data on both biomarkers and self‐reports were used in this estimation. The parameter estimates and their estimated standard errors are shown in Table I. Note that *μ*_*Ti*_ refers to the expected value of *T*_*ij*_, which, in this example, is the true intake to a power of 0.3. The estimated intervention effect from this analysis is ${\hat{\mu }}_{T2}-{\hat{\mu }}_{T1}=4.123-4.612=-0.489$, which has estimated standard error 0.038. There is some evidence that the error in the self‐reports is differential; the *p*‐value from a joint test of the null hypothesis that *α*_11_ = *α*_12_ and *α*_01_ = *α*_02_ is 0.022. Espeland *et al*. 22 studied data from the TONE study and also found evidence of differential error in the self‐reports.

**Table I:** Estimates of model parameters in the TONE data.

Parameter	Estimate	SE
Group 1		
*μ*_*T*1_	4.612	0.025
$\sigma_{T1}^{2}$	0.123	0.018
$\sigma_{M1}^{2}$	0.205	0.015
*α*_01_	0.287	0.640
*α*_11_	0.799	0.139
$\sigma_{Q1}^{2}$	0.300	0.027
Group 2		
*μ*_*T*2_	4.123	0.029
$\sigma_{T2}^{2}$	0.205	0.025
$\sigma_{M2}^{2}$	0.246	0.018
*α*_02_	1.534	0.349
*α*_12_	0.494	0.084
$\sigma_{Q2}^{2}$	0.284	0.023

TONE, Trial of Nonpharmacologic Intervention in the Elderly; SE, standard error.

To investigate the methods for estimating the intervention effect that make use of the self‐reports, we considered scenarios in which the biomarker measures were available only in a subgroup of the participants. We considered subgroups comprising 10%, 25%, 50%, and 100% of the study population. We selected individuals at random from the study population and set the biomarker measurements of those not selected to be missing. We estimated the intervention effect using biomarkers only and using biomarkers and self‐reports combined, the latter using both the method of moments Buonaccorsi approach and maximum likelihood, each under differential and non‐differential error. The results are shown in Table II.

**Table II:** Results from the TONE data.

Method	Percentage of individuals in the calibration sub‐study			
	10%	25%	50%	100%
Using method of moments				
Using biomarkers only	−0.666 (0.138)	−0.599 (0.076)	−0.550 (0.053)	−0.489 (0.038)
Combined: differential error	−0.666 (0.138)	−0.572 (0.075)	−0.549 (0.051)	—
Combined: non‐differential error	−0.705 (0.109)	−0.606 (0.074)	−0.565 (0.052)	−0.498 (0.038)
Using maximum likelihood				
Using biomarkers only	−0.666 (0.131)	−0.597 (0.075)	−0.550 (0.053)	−0.489 (0.038)
Combined: differential error	−0.652 (0.122)	−0.590 (0.071)	−0.547 (0.051)	−0.489 (0.038)
Combined: non‐differential error	−0.739 (0.104)	−0.599 (0.066)	−0.554 (0.048)	−0.503 (0.037)

The estimated intervention effect and its estimated standard error (in brackets) using six approaches: using biomarkers only (estimated using method of moments and maximum likelihood) and using biomarkers and self‐reports combined using the method of moments Buonaccorsi approach and maximum likelihood. The combined estimates were obtained under the assumptions of differential or non‐differential error in the self‐reports. TONE, Trial of Nonpharmacologic Intervention in the Elderly.

Using method of moments estimation in the scenario with 10% of individuals in the calibration sub‐study, we found the same estimated intervention effect with the same standard error using the biomarkers data only as using the biomarkers and self‐reports combined using the Buonaccorsi approach under differential error. As the proportion of individuals in the calibration sub‐study increases, these two approaches continue to give similar estimates and standard errors. These results are not unexpected based on the theory in Section 3.1, where we showed that when there is differential error in the self‐report measurements, there may be little or nothing to gain from using the self‐reports in addition to the biomarkers. It is interesting that the self‐reports appear to add nothing even when the proportion of individuals having the biomarker measurements is small. However, as shown in the simulation studies (in the succeeding discussion), there are cases with finite sample sizes where self‐reports do add information, even when the measurement error is differential.

The intervention effect estimates obtained using maximum likelihood estimation are close to the method of moments estimates. The standard errors of the estimates using the biomarkers only and using the self‐reports allowing for differential error are slightly smaller than those obtained using method of moments when the proportion with biomarkers is small. However, when the proportion reaches 50%, the results from maximum likelihood and method of moments are nearly identical. Using maximum likelihood, there is a small reduction in the standard errors by including the self‐reports when the proportion with biomarkers is small, but this disappears as the proportion increases.

We have so far focused on the results obtained using biomarkers only or using the self‐report assuming differential error. Because there is evidence that the error is indeed differential, we would expect estimates of the intervention effect obtained under an assumption of non‐differential error to be biased. In fact, the estimated intervention effect under the non‐differential error assumption is indeed larger than that found under the differential error assumption or using biomarkers only. In the simulation study, we investigate the bias from wrongly assuming non‐differential error in the self‐reports.

## 5 Simulation study

### 5.1 Simulating the data

We used simulation to investigate the issues raised in the preceding discussion, including those raised by the results from the example. The simulation study was based on the TONE data. We generated data for a study population of 1000 individuals, with 500 in each of the two treatment groups. For each individual, the true outcome measure *T* was generated according to model (1), a single self‐report measurement *Q* was generated according to model (2), and two biomarker measurements, *M*_1_ and *M*_2_, were generated according to model (3).

The parameters used in the simulation study approximately followed the values found in the TONE study and given in Table I. We used *μ*_*T*1_ = 4.6, *μ*_*T*2_ = 4.1 across all simulation scenarios, giving an intervention effect of −0.5. We chose the *α*‐parameters of model (2) as follows:

*Differential error*: *α*_01_ = 0.3, *α*_11_ = 0.8,  *α*_02_ = 1.5,  *α*_12_ = 0.5.
*Non‐differential error*: *α*_01_ = *α*_02_ = *α*_0_ = 0.9, *α*_11_ = *α*_12_ = *α*_1_ = 0.65.

We used a common variance in true intake in the two treatment groups, $\sigma_{T1}^{2}=\sigma_{T2}^{2}=0.1$, and in the errors in the biomarkers, $\sigma_{M1}^{2}=\sigma_{M2}^{2}=0.2$. We varied the variance in the errors in self‐reports across simulations but set it to be the same in the two treatment groups ( $\sigma_{Q1}^{2}=\sigma_{Q2}^{2}=0.09,\;0.3,\;0.5,\;0.7$). When the value is 0.09, the reliability for the self‐reports averaged over the two groups is approximately the same as the 0.5 reliability for the biomarkers.

The differential and non‐differential error scenarios and the four values of error variance in the self‐reports resulted in a total of eight scenarios. In each of these, we also varied the percentage of individuals in the calibration sub‐study, that is, with the biomarker measurements available: 10%, 25%, 50%, and 100%. Both biomarker measurements were set to be missing for individuals not in the calibration sub‐study.

For each simulated data set, we estimated the intervention effect using three method of moments approaches: (i) using biomarkers only; (ii) using the combined estimate obtained using the Buonaccorsi method assuming differential error; and (iii) using the combined estimate obtained using the Buonaccorsi method assuming non‐differential error. Note that only one of the combined estimates uses the assumption that corresponds to the way the data were generated. We also estimated the intervention effect using maximum likelihood.

For each scenario, we generated 1000 simulated data sets, and for each method, we calculated the average bias in the intervention effect estimate, its mean squared error, its empirical standard deviation, the square root of its mean model‐based variance, the coverage of its 95% confidence intervals, and its efficiency relative to the ‘gold standard’ situation where the biomarkers are observed for all individuals and the method of moments or maximum likelihood applied.

We performed two additional simulations in which the true intake is not normally distributed and therefore dietary measurements do not follow a multivariate normal distribution. These are described in Appendix S3 and illustrate the use of sandwich estimates for the variances obtained under the maximum likelihood analysis.

In the Supporting Information (see Appendix S4 and additional text files), we provide example R code that can be used to simulate data as described previously and implement the methods described in this paper.

### 5.2 Simulation study results

The results for the method of moments approach are shown in Tables III and IV. The corresponding results obtained for maximum likelihood estimation were very similar and are shown in the Tables S1 and S2.

**Table III:** Simulation study results for data simulated under differential error.

Method	Bias	MSE	Emp SD	Model SE	Cov	Eff
(a) $\sigma_{Q1}^{2}=\sigma_{Q2}^{2}=0.09$						
Calibration sub‐study: 10%						
Biomarkers only	0.002	0.008	0.087	0.089	95.6	11.3
Combined: differential error [998]*	0.001	0.005	0.074	0.075	94.9	15.7
Combined: non‐differential error	−0.035	0.007	0.075	0.073	92.5	15.4
Calibration sub‐study: 25%						
Biomarkers only	0.001	0.003	0.056	0.057	95.0	27.4
Combined: differential error	−0.000	0.002	0.048	0.048	94.4	37.2
Combined: non‐differential error	−0.034	0.003	0.048	0.048	88.3	37.5
Calibration sub‐study: 50%						
Biomarkers only	−0.000	0.002	0.041	0.040	94.0	50.4
Combined: differential error	−0.001	0.001	0.037	0.036	93.9	62.3
Combined: non‐differential error	−0.031	0.002	0.037	0.036	85.1	63.7
Calibration sub‐study: 100%						
Biomarkers only	−0.000	0.001	0.029	0.028	94.4	100.0
Combined: differential error	—	—	—	—	—	—
Combined: non‐differential error	−0.024	0.001	0.027	0.027	85.4	114.6
(b) $\sigma_{Q1}^{2}=\sigma_{Q2}^{2}=0.3$						
Calibration sub‐study: 10%						
Biomarkers only	0.002	0.008	0.087	0.089	95.6	11.3
Combined: differential error [997]*	0.002	0.007	0.085	0.085	95.6	11.9
Combined: non‐differential error	−0.019	0.006	0.077	0.078	95.0	14.4
Calibration sub‐study: 25%						
Biomarkers only	0.001	0.003	0.056	0.057	95.0	27.4
Combined: differential error	−0.000	0.003	0.054	0.054	94.8	29.5
Combined: non‐differential error	−0.021	0.003	0.049	0.051	93.3	35.1
Calibration sub‐study: 50%						
Biomarkers only	−0.000	0.002	0.041	0.040	94.0	50.4
Combined: differential error	−0.000	0.002	0.040	0.039	94.0	53.8
Combined: non‐differential error	−0.020	0.002	0.038	0.037	91.4	60.6
Calibration sub‐study: 100%						
Biomarkers only	−0.000	0.001	0.029	0.028	94.4	100.0
Combined: differential error	—	—	—	—	—	—
Combined: non‐differential error	−0.014	0.001	0.028	0.027	91.6	111.1
(c) $\sigma_{Q1}^{2}=\sigma_{Q2}^{2}=0.5$						
Calibration sub‐study: 10%						
Biomarkers only	0.002	0.008	0.087	0.089	95.6	11.3
Combined: differential error [997]*	0.002	0.008	0.087	0.088	95.9	11.3
Combined: non‐differential error	−0.006	0.007	0.081	0.082	95.5	13.1
Calibration sub‐study: 25%						
Biomarkers only	0.001	0.003	0.056	0.057	95.0	27.4
Combined: differential error	0.000	0.003	0.056	0.056	94.7	27.9
Combined: non‐differential error	−0.011	0.003	0.052	0.053	94.5	32.1
Calibration sub‐study: 50%						
Biomarkers only	−0.000	0.002	0.041	0.040	94.0	50.4
Combined: differential error	−0.000	0.002	0.041	0.040	93.7	51.6
Combined: non‐differential error	−0.011	0.002	0.039	0.038	93.6	56.7
Calibration sub‐study: 100%						
Biomarkers only	−0.000	0.001	0.029	0.028	94.4	100.0
Combined: differential error	—	—	—	—	—	—
Combined: non‐differential error	−0.008	0.001	0.028	0.028	93.5	106.5
(d) $\sigma_{Q1}^{2}=\sigma_{Q2}^{2}=0.7$						
Calibration sub‐study: 10%						
Biomarkers only	0.002	0.008	0.087	0.089	95.6	11.3
Combined: differential error [997]*	0.002	0.008	0.087	0.088	95.8	11.2
Combined: non‐differential error	0.001	0.007	0.084	0.084	94.8	12.3
Calibration sub‐study: 25%						
Biomarkers only	0.001	0.003	0.056	0.057	95.0	27.4
Combined: differential error	0.000	0.003	0.056	0.056	94.5	27.5
Combined: non‐differential error	−0.005	0.003	0.053	0.054	94.5	30.2
Calibration sub‐study: 50%						
Biomarkers only	−0.000	0.002	0.041	0.040	94.0	50.4
Combined: differential error	−0.000	0.002	0.041	0.040	93.7	50.9
Combined: non‐differential error	−0.006	0.002	0.040	0.039	94.1	54.1
Calibration sub‐study: 100%						
Biomarkers only	−0.000	0.001	0.029	0.028	94.4	100.0
Combined: differential error	—	—	—	—	—	—
Combined: non‐differential error	−0.005	0.001	0.029	0.028	94.2	103.8

Results are shown from three analysis methods: using the biomarker data only and using the biomarker and self‐reports combined under the correct assumption of differential error in the self‐reports and under the incorrect assumption of non‐differential error. All estimates were obtained using method of moments, and the combined estimates were obtained using the Buonaccorsi approach. Results are shown separately for different values of the error variability in the self‐reports ( $\sigma_{Q1}^{2}=\sigma_{Q2}^{2}$) and for different proportions of individuals in the calibration sub‐study.

Bias, average bias in the intervention effect estimate across 1000 simulations; MSE, mean squared error of the intervention effect estimate across 1000 simulations; Emp SD, standard deviation of the 1000 intervention effect estimates; Model SE, square‐root of the mean of the variances of the 1000 intervention effect estimates; Cov, percentage of the 1000 95% confidence intervals for the intervention effect, which contained the true value; Eff, ratio of the variance of the 1000 intervention effect estimates obtained when the biomarkers are available in 100% of individuals to the variance of the 1000 intervention effect estimates from a given method, expressed as a percentage.

* In the case of a small calibration sub‐study, a small number of simulations resulted in negative variance estimates. In these situations, the number of simulations on which the results are based is given in square brackets.

**Table IV:** Simulation study results for data simulated under non‐differential error.

Method	Bias	MSE	Emp SD	Model SE	Cov	Eff
(a) $\sigma_{Q1}^{2}=\sigma_{Q2}^{2}=$ 0.09						
Calibration sub‐study: 10%						
Biomarkers only	0.002	0.008	0.087	0.089	95.6	11.3
Combined: differential error [998]*	0.001	0.005	0.073	0.074	94.9	16.1
Combined: non‐differential error	0.002	0.005	0.068	0.070	95.7	18.8
Calibration sub‐study: 25%						
Biomarkers only	0.001	0.003	0.056	0.057	95.0	27.4
Combined: differential error	−0.000	0.002	0.048	0.048	94.8	37.7
Combined: non‐differential error	0.001	0.002	0.045	0.046	96.1	43.3
Calibration sub‐study: 50%						
Biomarkers only	−0.000	0.002	0.041	0.040	94.0	50.4
Combined: differential error	−0.000	0.001	0.037	0.036	94.0	62.6
Combined: non‐differential error	−0.000	0.001	0.035	0.035	94.4	68.8
Calibration sub‐study: 100%						
Biomarkers only	−0.000	0.001	0.029	0.028	94.4	100.0
Combined: differential error	—	—	—	—	—	—
Combined: non‐differential error	−0.001	0.001	0.028	0.027	94.8	111.3
(b) $\sigma_{Q1}^{2}=\sigma_{Q2}^{2}=0.3$						
Calibration sub‐study: 10%						
Biomarkers only	0.002	0.008	0.087	0.089	95.6	11.3
Combined: differential error [997]*	0.002	0.007	0.084	0.085	95.6	12.1
Combined: non‐differential error	0.007	0.006	0.076	0.077	94.8	14.9
Calibration sub‐study: 25%						
Biomarkers only	0.001	0.003	0.056	0.057	95.0	27.4
Combined: differential error	−0.000	0.003	0.054	0.054	94.7	29.9
Combined: non‐differential error	0.003	0.002	0.049	0.050	95.8	35.3
Calibration sub‐study: 50%						
Biomarkers only	−0.000	0.002	0.041	0.040	94.0	50.4
Combined: differential error	−0.000	0.002	0.040	0.039	93.9	54.2
Combined: non‐differential error	0.001	0.001	0.038	0.037	94.3	59.3
Calibration sub‐study: 100%						
Biomarkers only	−0.000	0.001	0.029	0.028	94.4	100.0
Combined: differential error	—	—	—	—	—	—
Combined: non‐differential error	−0.000	0.001	0.028	0.028	94.8	106.0
(c) $\sigma_{Q1}^{2}=\sigma_{Q2}^{2}=0.5$						
Calibration sub‐study: 10%						
Biomarkers only	0.002	0.008	0.087	0.089	95.6	11.3
Buonaccorsi: differential error [997]*	0.002	0.008	0.087	0.088	95.9	11.4
Buonaccorsi: non‐differential error	0.012	0.007	0.082	0.081	93.8	12.7
Calibration sub‐study: 25%						
Biomarkers only	0.001	0.003	0.056	0.057	95.0	27.4
Buonaccorsi: differential error	0.000	0.003	0.055	0.056	94.7	28.0
Buonaccorsi: non‐differential error	0.005	0.003	0.053	0.053	95.1	30.8
Calibration sub‐study: 50%						
Biomarkers only	−0.000	0.002	0.041	0.040	94.0	50.4
Buonaccorsi: differential error	−0.000	0.002	0.041	0.039	93.8	51.8
Buonaccorsi: non‐differential error	0.001	0.002	0.040	0.038	93.8	54.4
Calibration sub‐study: 100%						
Biomarkers only	−0.000	0.001	0.029	0.028	94.4	100.0
Buonaccorsi: differential error	—	—	—	—	—	—
Buonaccorsi: non‐differential error	−0.000	0.001	0.029	0.028	95.0	103.1
(d) $\sigma_{Q1}^{2}=\sigma_{Q2}^{2}=0.7$						
Calibration sub‐study: 10%						
Biomarkers only	0.002	0.008	0.087	0.089	95.6	11.3
Buonaccorsi: differential error [997]*	0.002	0.008	0.087	0.088	95.7	11.3
Buonaccorsi: non‐differential error	0.016	0.008	0.086	0.083	93.0	11.5
Calibration sub‐study: 25%						
Biomarkers only	0.001	0.003	0.056	0.057	95.0	27.4
Buonaccorsi: differential error	0.000	0.003	0.056	0.056	94.5	27.5
Buonaccorsi: non‐differential error	0.006	0.003	0.055	0.054	94.9	28.6
Calibration sub‐study: 50%						
Biomarkers only	−0.000	0.002	0.041	0.040	94.0	50.4
Buonaccorsi: differential error	−0.000	0.002	0.041	0.040	93.6	51.0
Buonaccorsi: non‐differential error	0.002	0.002	0.041	0.039	93.7	52.2
Calibration sub‐study: 100%						
Biomarkers only	−0.000	0.001	0.029	0.028	94.4	100.0
Buonaccorsi: differential error	—	—	—	—	—	—
Buonaccorsi: non‐differential error	−0.000	0.001	0.029	0.028	95.0	101.8

Results are shown from three analysis methods: using the biomarker data only and using the biomarker and self‐reports combined under the assumption of differential error in the self‐reports and under the correct assumption of non‐differential error. All estimates were obtained using method of moments and the combined estimates were obtained using the Buonaccorsi approach. Results are shown separately for different values of the error variability in the self‐reports ( $\sigma_{Q1}^{2}=\sigma_{Q2}^{2}$) and for different proportions of individuals in the calibration sub‐study.

Bias, average bias in the intervention effect estimate across 1000 simulations; MSE, mean squared error of the intervention effect estimate across 1000 simulations; Emp SD, standard deviation of the 1000 intervention effect estimates; Model SE, square‐root of the mean of the variances of the 1000 intervention effect estimates; Cov, percentage of the 1000 95% confidence intervals for the intervention effect, which contained the true value; Eff, ratio of the variance of the 1000 intervention effect estimates obtained when the biomarkers are available in 100% of individuals to the variance of the 1000 intervention effect estimates from a given method, expressed as a percentage.

* In the case of a small calibration sub‐study, a small number of simulations resulted in negative variance estimates. In these situations, the number of simulations on which the results are based is given in square brackets.

Table III shows results when the error in the self‐report was differential. As expected, unbiased estimates of the treatment effect were obtained from the analysis that used only the biomarker measurements (‘Biomarkers only’) and the analysis that additionally used the self‐reports with an assumption of differential error (‘Combined: differential error’), which is how the self‐report data were generated. The coverage of the estimates obtained under these two methods was at the nominal level. The analysis that used both biomarkers and self‐reports but assumed non‐differential error (‘Combined: non‐differential error’), contrary to how the data were generated, resulted in biased estimates of the intervention effect and poor coverage.

The percentage efficiency of the estimated intervention effect in a study using only the biomarker data in the calibration sub‐study relative to the ‘gold‐standard’ situation in which biomarker measurements were available in the full sample was approximately equal to the percentage of individuals in the calibration sub‐study: 10%, 25%, and 50% in the calibration sub‐study gave relative efficiencies of 11.3%, 27.4%, and 50.4%, respectively.

When the reliability of the self‐reports was similar to that of the biomarkers, that is, when the error variance of the self‐report was relatively low ( $\sigma_{Q1}^{2}=\sigma_{Q2}^{2}=0.09$) (Table III(a)), there was a gain in efficiency from using the self‐reports in addition to the biomarker measurements. When the calibration sub‐study contained 10% of individuals, the relative efficiency increased from 11.3% using the biomarker data only to 15.7% when additionally making use of the self‐report data through the Buonaccorsi approach. When the calibration sub‐study contained 25% of individuals, the relative efficiency increased from 27.4% to 37.2%, and when the calibration sub‐study contained 50% of individuals, the relative efficiency increased from 50.4% to 62.3%. The results obtained using maximum likelihood (Table S1) showed that there is nothing to be gained by using the self‐reports when biomarker data are available for the complete study sample. Note that when all individuals have biomarker data, the Buonaccorsi‐combined estimate is identical to the biomarkers estimate.

The aforementioned results refer to the situation in which $\sigma_{Q1}^{2}=\sigma_{Q2}^{2}=0.09$ (Table III(a)). When the reliability of the self‐reports decreased relative to the biomarkers, with an error variance of the self‐report equal to 0.3 (Table III(b)), which was the estimated value obtained in the TONE example, there was minimal gain in efficiency from using the self‐reports in addition to the biomarkers. For example, with 10% of individuals in the calibration sub‐study, the efficiency of the intervention effect estimate relative to the gold‐standard increased from 11.3% using the biomarker data to only 11.9% when the self‐reports were additionally used. The efficiency gain declined further as the error variance of the self‐reports increased (Tables III(c) and (d)). In summary, the simulation results showed that if the error variance of the self‐report is high relative to that in the biomarker measure, or in other words the reliability of the self‐report is low relative to the biomarker, there is little to be gained from using the self‐report data available on all individuals over using just the biomarker data on a (possibly small) subset of those individuals.

Table IV shows the results when the error in the self‐report was non‐differential. In this scenario, all three analysis methods (‘Biomarkers only’, ‘Combined: differential error’, and ‘Combined: non‐differential error’) gave unbiased estimates of the intervention effect and correct coverage. Note that we expect the combined estimate assuming differential error to give unbiased estimates because non‐differential error may be considered a special case of differential error. The combined estimate obtained using the Buonaccorsi approach that uses the self‐report data in addition to the biomarker data and assumes non‐differential error in the self‐report gave a gain in precision in the intervention effect estimate relative to the analysis using the biomarker data only. With a calibration sub‐study containing 10% of the study sample, and error variance $\sigma_{Q1}^{2}=\sigma_{Q2}^{2}=0.09$, the relative efficiency of the intervention effect estimate increased from 11.3% when using the biomarker data only to 18.8% when using the combined estimate under the correct assumption of non‐differential error (Table IV(a)). The relative gain in efficiency was smaller but still appreciable when the error variance in the self‐report was larger, representing a lower reliability of the self‐reports (Table IV(b)). This gain in efficiency by making use of self‐reports is supported by the theory presented in Section 3.1. Assuming differential error when it was not necessary resulted in a smaller gain, which was similar to that seen under differential error (Table III). Gains in efficiency from the combined estimate became very small when the error variance increased beyond 0.3 under both methods.

There was little or nothing to be gained by estimating the intervention effect using maximum likelihood instead of the method of moments, neither under differential nor non‐differential error in the self‐report (Tables S1 and S2).

Our findings from the simulation study have important implications for the design of dietary intervention trials, which we discuss in the next section.

## 6 Discussion and recommendations

Although the issue of dietary measurement error has received much attention in relation to observational studies in nutritional epidemiology, there is relatively little written on its impact in dietary intervention studies in which the main interest is in the achievement of dietary change. In view of several reports that the measurement error in self‐reported intakes in dietary intervention studies is differential (e.g., 16 and 22) and the expectation that participants who are encouraged by the investigators to change their diet will report differently from those not so encouraged, it seems inadvisable to base evaluation of interventions solely on self‐report data. It would seem that whenever possible, objective data on dietary intake should be collected. In this paper, we have assumed that a biomarker that provides an unbiased assessment of an individual's dietary intake is available. Further on, we discuss the options when an unbiased biomarker measurement is not available.

However, collection of such biomarker data often is very expensive, as in the case of doubly labeled water for measuring energy intake 23, or imposes a high burden on the participant, as in the case 24‐h urinary collections. Therefore, it is important to know in what circumstances the less expensive, less burdensome collection of self‐report data can be a helpful addition to the objective data, for example, by allowing the collection of biomarker measurements on a subsample rather than the full sample. We have shown that even though such reports provide biased measures of dietary intake, they can add information about the intervention effect when used in combination with objective biomarker data. A situation in which they are helpful is when (a) the biomarker is measured in a subset of participants and the self‐report in all participants and (b) the reliability of the self‐report is comparable with or better than the biomarker. Although the theory suggests that asymptotically the information from the self‐reports will add little to that from biomarkers alone when the error in self‐reports is differential (Section 3.1), our simulations showed that for finite sample sizes, there is still something to be gained by using the self‐report data under the aforementioned situation (b).

It is important to note here that the self‐report may be defined as the average of several administrations of an instrument (e.g., multiple 24‐h recalls), in which case the reliability of the self‐report may be made greater than that of data from a single administration of the instrument. For example, in TONE, the reliability for a single 24‐h recall was only 0.26 (in Group 1) compared with 0.60 for the biomarker, but if four repeats of a 24‐h recall were used, the reliability of the self‐report would increase to 0.59, close to that of the biomarker. This has important implications for study design decisions. Using the results of the first part of Table III ( $\sigma_{Q1}^{2}=\sigma_{Q2}^{2}=0.09$; final column), where the reliabilities of the self‐report and biomarker are also approximately equal, 100 persons with self‐report plus biomarker measurements and another 900 with just self‐report would have similar efficiency to a design with 157 persons with biomarkers only; a design having 250 persons with self‐report and biomarkers plus another 750 with self‐report only would be about as efficient as 372 persons with biomarkers only; and a design having 500 persons with self‐report and biomarkers plus another 500 with self‐report only would be about as efficient as 623 persons with biomarkers only. Clearly, cost issues and statistical power calculations would need to be factored in to arrive at the best combination, but it is clear that there could be a role for the use of self‐report data. It would be helpful to develop a sample size program, based on the Buonaccorsi method of analysis, that includes the costs of entering and assessing a participant who completes a self‐report and biomarker relative to one who completes a self‐report only.

Both theory and simulation results showed that self‐reports can add information to objective biomarkers when the measurement error in the self‐report is non‐differential, that is, the same in each comparative group. The benefits of using the self‐reports persisted for larger error variances in this situation compared with when the error was differential. The non‐differential situation is less likely to apply in behavioral interventions compared with other types of interventions but may hold when the experimental intervention does not involve close contact between the investigators and the participants, or when the target of the intervention is not diet in itself but rather relates to shifts to environments that are meant to support healthier eating. For example, interventions that involve provision of extra food coupons, allowing purchase of healthier foods, may not induce differences between the intervention and control group in the accuracy with which they report their intake.

An interesting finding in our simulations was that the methods of moments estimates obtained using Buonaccorsi's method appeared almost as efficient as the MLEs. One advantage of the Buonaccorsi approach is that, before combination, it naturally provides separate estimates of the intervention effect, one based only upon the biomarker data and one based also on the self‐report data, each having its own standard error. This enables the investigator to appreciate the contribution of each type of measurement to the overall estimate of the intervention effect.

The main limitation of the work that we have presented is that the cases in which an unbiased biomarker of dietary intake is available are currently limited (including only sodium, potassium, protein, and energy studies). Work is ongoing to widen the class of unbiased dietary biomarkers 24. How best to design studies without the inclusion of such an unbiased biomarker is a matter for further research. However, progress in this area may come from the availability of concentration biomarkers, for which the association between intake and serum or blood levels are known from feeding studies 25. A concentration biomarker is one that is correlated with dietary intake but does not provide an unbiased measure. There are many examples of such biomarkers, such as serum lipids and serum carotenoids, many of which are correlated with intakes of dietary components that may be targets of interventions. When data from feeding studies with these biomarkers are available, it may be possible via statistical models to translate the biomarker levels into levels of usual intake, as has been performed previously with the carotenoids lutein and zeaxanthin 26. With such ‘translation’, the concentration biomarker could then take the place of the unbiased biomarker that we have assumed in this paper.

Methods for correcting for the impact of measurement error in self‐report dietary data in studies of intervention effects on dietary outcome have so far received relatively little attention in the large literature on dietary measurement error, where the focus has been instead been on dietary intake as an exposure. In this paper, we have outlined methods for error correction when dietary intake is an outcome in an intervention trial when self‐report data are available in the main study and biomarker data are available in a calibration study. The methods described include those for handling differential error in self‐report data, which may be common. Using both theoretical and simulation results, we have outlined situations in which self‐report data in addition to biomarker data can contribute to improve intervention effect estimates, and others in which, perhaps surprisingly, self‐report data may contribute little. Further work is needed to develop a tool for investigators planning dietary intervention trials that incorporates information on the cost of obtaining self‐report and biomarker data in addition to information on the relative reliability of the two instruments.

## Supporting information

Supporting info item

Supporting info item

Supporting info item

Supporting info item

Supporting info item

Supporting info item

Supporting info item

Supporting info item

Supporting info item

Supporting info item

Supporting info item

Supporting info item
