# Supplementary material for: Statistical issues related to dietary intake as the response variable in intervention trials
Source: Stat Med. 2016 Jun 20;35(25):4493–508. doi: 10.1002/sim.7011 (PMC5050089; doi:10.1002/sim.7011)
Supplement: Supplementary file 1 — Supporting info item [file SIM-35-4493-s001.pdf]

Web Appendix: Statistical issues related to dietary intake as the response variable in intervention trials

Appendix S1. Combination of estimates for the Buonaccorsi approach

Weighted combination of $\hat{\theta}_{(i)}$ and $\hat{\theta}_{(ii)}$

A weighted variance combined estimate of $\hat{\theta}_{(i)}$ and $\hat{\theta}_{(ii)}$, denoted, $\hat{\theta}_c$ is given by

$$\hat{\theta}_c = (\mathbf{1}^T\hat{\Sigma}\mathbf{1})^{-1}\mathbf{1}^T\hat{\Sigma}^{-1}\left(\hat{\theta}_{(i)} \quad \hat{\theta}_{(ii)}\right)' \qquad \text{(A1)}$$

where $\mathbf{1} = \begin{pmatrix}1\\1\end{pmatrix}$ is a vector of 1s and $\hat{\Sigma} = \begin{pmatrix}\mathrm{var}(\hat{\theta}_{(i)}) & \mathrm{cov}(\hat{\theta}_{(i)}, \hat{\theta}_{(ii)})\\ \mathrm{cov}(\hat{\theta}_{(i)}, \hat{\theta}_{(ii)}) & \mathrm{var}(\hat{\theta}_{(ii)})\end{pmatrix}$ is the estimated variance covariance matrix for $\hat{\theta}_{(i)}$ and $\hat{\theta}_{(ii)}$. The expression in (A1) reduces to

$$\hat{\theta}_c = \frac{\hat{\theta}_{(ii)}\mathrm{var}\left(\hat{\theta}_{(i)}\right) + \hat{\theta}_{(i)}\mathrm{var}\left(\hat{\theta}_{(ii)}\right) - \ (\hat{\theta}_{(i)} + \hat{\theta}_{(ii)})\mathrm{cov}(\hat{\theta}_{(i)}, \hat{\theta}_{(ii)})}{\mathrm{var}\left(\hat{\theta}_{(i)}\right)+\mathrm{var}\left(\hat{\theta}_{(ii)}\right) + 2\mathrm{cov}(\hat{\theta}_{(i)}, \hat{\theta}_{(ii)})} \qquad \text{(A2)}$$

The estimates $\hat{\theta}_{(i)}$ and $\hat{\theta}_{(ii)}$ are functions of estimates of the parameters $\mu_{Mi}, \mu_{Qi}, \mu_{Qi}^{(s)}, \alpha_{0i}, \alpha_{1i}$ $(i = 1{,}2)$, where $\mu_{Qi}^{(s)}$ denotes the expected value of the self-reports in the sub-study and in treatment group i.Therefore we require estimates of their variance covariance matrix to obtain the estimated variance covariance matrix $\hat{\Sigma}$. We do this using an estimating equations approach.

Estimating equations approach

We begin by giving a general description of the estimating equations approach. Suppose we have observed data Y_k on N $(k = 1, \ldots, N)$ individuals which is to be used to estimate a vector of parameters Θ. The estimating equations for Θ are those whose solutions are the estimates $\hat{\Theta}$:

$$N^{-1}\sum_{k=1}^{N}\Psi_k(Y_k, \Theta) = 0 \qquad \text{(A3)}$$

where the $\Psi_k$are vectors of known functions. An estimate of the variance of $\hat{\Theta}$ is

$$\mathrm{var}\left(\hat{\Theta}\right) = N^{-1}\hat{A}_N^{-1}\hat{B}_N\left(\hat{A}_N^{-1}\right)' \qquad \text{(A4)}$$

where

$$A_N = N^{-1}\sum_{k=1}^{n}\frac{d}{d\Theta}\Psi_k(Y_k,\Theta) \quad \text{(A5)}$$

and

$$B_N = N^{-1}\sum_{k=1}^{N}\Psi_k(Y_k,\Theta)\Psi_k(Y_k,\Theta)' \quad \text{(A6)}$$

and $\hat{A}_N$ and $\hat{B}_N$ are A_N and B_N respectively evaluated at $\widehat{\Theta}$.

Differential error

The estimating equations for parameters $\mu_{Mi}, \mu_{Qi}, \mu_{Qi}^{(s)}, \alpha_{0i}, \alpha_{1i}$ $(i = 1,2)$ are respectively

$$N_i^{-1}\sum_{j=1}^{N_i}\left(\bar{M}_{ij} - \mu_{Mi}\right) I_{ij}^{(s)} N_i/n_i = 0 \quad \text{(A7)}$$

$$N_i^{-1}\sum_{j=1}^{N_i}\left(Q_{ij} - \mu_{Qi}\right) = 0 \quad \text{(A8)}$$

$$N_i^{-1}\sum_{j=1}^{N_i}\left(Q_{ij} - \mu_{Qi}^{(s)}\right) I_{ij}^{(s)} N_i/n_i = 0 \quad \text{(A9)}$$

$$N_i^{-1}\sum_{j=1}^{N_i}(Q_{ij} - \alpha_{0i} - \alpha_{1i}M_{ij1}) I_{ij}^{(s)} N_i/n_i = 0 \quad \text{(A10)}$$

$$N_i^{-1}\sum_{j=1}^{N_i}(Q_{ij} - \alpha_{0i} - \alpha_{1i}M_{ij1}) M_{ij2} I_{ij}^{(s)} N_i/n_i = 0 \quad \text{(A11)}$$

where $I_j^{(s)}$ is an indicator taking value 1 if individual j is in the calibration sub-study and value 0 otherwise. The biomarker data M_{ijk} are only observed in the sub-study $(j = 1, \ldots, n_i)$ and so we may assume the biomarker values to take value 0 (or any other value) outside the sub-study for the purposes of the above equations.

Using the delta method it can be shown that the variances of $\hat{\theta}_{(\mathrm{i})}$ and $\hat{\theta}_{(\mathrm{ii})}$ and their covariance are given by:

$$\mathrm{var}\left(\hat{\theta}_{(\mathrm{i})}\right) = \mathrm{var}(\hat{\mu}_{M2}) + \mathrm{var}(\hat{\mu}_{M1}) \quad \text{(A12)}$$

$$\mathrm{var}\left(\hat{\theta}_{(\mathrm{ii})}\right) \approx \mathrm{var}\left(\theta_{(\mathrm{i})}\right) + \sum_{i=1}^{2} \frac{1}{\hat{\alpha}_{1i}} \left\{ \mathrm{var}\left(\hat{\mu}_{Qi}\right) + \mathrm{var}\left(\hat{\mu}_{Qi}^{(s)}\right) - 2\mathrm{cov}\left(\hat{\mu}_{Qi}, \hat{\mu}_{Qi}^{(s)}\right) \right\} \quad \text{(A13)}$$

$$+ \sum_{i=1}^{2} \left(\frac{\hat{\mu}_{Qi} - \hat{\mu}_{Qi}^{(s)}}{\hat{\alpha}_{1i}^{2}} \right)^{2} \mathrm{var}\left(\hat{\alpha}_{1i}\right)$$

$$- 2 \sum_{i=1}^{2} \left(\frac{\hat{\mu}_{Qi} - \hat{\mu}_{Qi}^{(s)}}{\hat{\alpha}_{1i}^{3}} \right) \left\{ \mathrm{cov}\left(\hat{\mu}_{Qi}, \hat{\alpha}_{1i}\right) - \mathrm{cov}\left(\hat{\mu}_{Qi}^{(s)}, \hat{\alpha}_{1i}\right) \right\}$$

$$+ \sum_{i=1}^{2} \frac{1}{\alpha_{1i}} \mathrm{cov}\left(\hat{\mu}_{Mi}, \hat{\mu}_{Qi}\right)$$

$$- \sum_{i=1}^{2} \frac{1}{\alpha_{1i}} \mathrm{cov}\left(\hat{\mu}_{Mi}, \hat{\mu}_{Qi}^{(s)}\right) - \sum_{i=1}^{2} \left(\frac{\hat{\mu}_{Qi} - \hat{\mu}_{Qi}^{(s)}}{\hat{\alpha}_{1i}} \right) \mathrm{cov}\left(\hat{\mu}_{Mi}, \hat{\alpha}_{1i}\right)$$

$$\mathrm{cov}\left(\hat{\theta}_{(\mathrm{i})}, \hat{\theta}_{(\mathrm{ii})}\right) \approx \mathrm{var}\left(\theta_{(\mathrm{i})}\right) \quad \text{(A14)}$$

$$+ \sum_{i=1}^{2} \frac{1}{\alpha_{1i}} \mathrm{cov}\left(\hat{\mu}_{Mi}, \hat{\mu}_{Qi}\right)$$

$$+ \sum_{i=1}^{2} \frac{1}{\alpha_{1i}} \mathrm{cov}\left(\hat{\mu}_{Mi}, \hat{\mu}_{Qi}^{(s)}\right) - \sum_{i=1}^{2} \left(\frac{\hat{\mu}_{Qi} - \hat{\mu}_{Qi}^{(s)}}{\hat{\alpha}_{1i}} \right) \mathrm{cov}\left(\hat{\mu}_{Mi}, \hat{\alpha}_{1i}\right)$$

Non-differential error

The estimating equations for parameters μ_{Mi}, μ_{Qi} $(i = 1,2)$ and α_0, α_1 are respectively

$$N^{-1} \sum_{k=1}^{N} (\bar{M}_k - \mu_{Mi}) I_k^{(s)} J_k^{(i)} N / n_i = 0 \quad \text{(A15)}$$

$$N^{-1} \sum_{k=1}^{N} (Q_k - \mu_{Mi}) J_k^{(i)} N / n_i = 0 \quad \text{(A16)}$$

$$N^{-1} \sum_{k=1}^{N} (Q_k - \alpha_0 - \alpha_1 M_{k1}) I_k^{(s)} N / (n_1 + n_2) = 0 \quad \text{(A17)}$$

$$N^{-1} \sum_{k=1}^{N} (Q_k - \alpha_0 - \alpha_1 M_{k1}) M_{k2} I_k^{(s)} N / (n_1 + n_2) = 0 \quad \text{(A18)}$$

where $J_k^{(i)}$ is an indicator taking value 1 if individual k is in treatment group i and value 0 otherwise, $I_k^{(s)}$ is an indicator taking value 1 if individual k is in the calibration sub-study and

value 0 otherwise, M_{k1} and M_{k2} are the first and second biomarker measurements for individual k $(k = 1, \dots, N)$.

The variance of the first estimate required for the Buonaccorsi approach, $\hat{\theta}_{(\mathrm{i})}$, is as given in (A10). The variance of the second estimate, $\hat{\theta}_{(\mathrm{ii})}$, is approximated by

$$\mathrm{var}\left(\hat{\theta}_{(\mathrm{ii})}\right) \approx \sum_{i=1}^{2} \left\{ \frac{1}{\hat{\alpha}_1^2} \mathrm{var}\left(\hat{\mu}_{Qi}\right) + \left(\frac{\hat{\mu}_{Qi}}{\hat{\alpha}_1^2}\right)^2 \mathrm{var}(\hat{\alpha}_1) - 2\frac{\hat{\mu}_{Qi}}{\hat{\alpha}_1^3} \mathrm{cov}\left(\hat{\mu}_{Qi}, \hat{\alpha}_1\right) \right\} - 2\left\{ \frac{1}{\hat{\alpha}_1^2} \mathrm{cov}\left(\hat{\mu}_{Q1}, \hat{\mu}_{Q2}\right) + \frac{\hat{\mu}_{Q1}\hat{\mu}_{Q2}}{\hat{\alpha}_1^4} \mathrm{var}(\hat{\alpha}_1) - \frac{\hat{\mu}_{Q1}}{\hat{\alpha}_1^3} \mathrm{cov}\left(\hat{\mu}_{Q2}, \hat{\alpha}_1\right) - \frac{\hat{\mu}_{Q2}}{\hat{\alpha}_1^3} \mathrm{cov}(\hat{\mu}_{Q1}, \hat{\alpha}_1) \right\} \tag{A19}$$

The covariance between $\hat{\theta}_{(\mathrm{i})}$ and $\hat{\theta}_{(\mathrm{ii})}$ is approximated by

$$\mathrm{cov}\left(\hat{\theta}_{(\mathrm{i})}, \hat{\theta}_{(\mathrm{ii})}\right) \approx \sum_{i=1}^{2} \frac{1}{\hat{\alpha}_1} \mathrm{cov}\left(\hat{\mu}_{Mi}, \hat{\mu}_{Qi}\right) - \sum_{i=1}^{2} \sum_{i'=1,2;i' \neq i} \frac{1}{\hat{\alpha}_1} \mathrm{cov}\left(\hat{\mu}_{Mi}, \hat{\mu}_{Qi'}\right) - \sum_{i=1}^{2} \frac{\hat{\mu}_{Qi}}{\hat{\alpha}_1^2} \mathrm{cov}(\hat{\mu}_{Mi}, \hat{\alpha}_1) + \sum_{i=1}^{2} \sum_{i'=1,2;i' \neq i} \frac{\hat{\mu}_{Qi}}{\hat{\alpha}_1^2} \mathrm{cov}(\hat{\mu}_{Mi'}, \hat{\alpha}_1) \tag{A20}$$

<u>Appendix S2: Sandwich estimate for the variance of MLEs under model mis-specification</u>

The sandwich estimate

The log likelihood of observed data Y_j on individuals $j = 1, \dots, n$ under a model $f(.)$ parametrized by θ is

$$l(\theta) = \sum_{j=1}^{n} l_j(\theta) = \sum_{j=1}^{n} f(Y_j;\theta) \tag{A21}$$

Maximum likelihood estimates for θ are obtained by as the values which maximise the likelihood, which are given by the solutions to $l'(\theta) = 0$, where $l'(\theta)$ denotes the vector of first derivatives of $l(\theta)$ with respect to θ. If the model $f(.)$ is correctly specified then the variance-covariance matrix for the maximum likelihood estimates $\hat{\theta}$ is estimated by the inverse of the information matrix (minus the matrix of 2nd derivatives of $l(\theta)$ with respect to θ; $-l''(\theta)$) evaluated at $\hat{\theta}$, which we denote $\hat{V}$. However, if $f(.)$ is mis-specified this estimate for the variance-covariance matrix for $\hat{\theta}$ is incorrect and an alternative is required. One option, as noted in the main text, is to use bootstrapping and this can be performed easily in standard software. Another option is to use the sandwich estimate. The sandwich variance estimate is given by $\hat{V}\widehat{W}\hat{V}$ where $\widehat{W}$ denotes the estimated variance-covariance matrix for the vector of first derivatives of the log likelihood $l'(\theta)$, evaluated at $\hat{\theta}$, which is given by

$$\widehat{W} = \sum_{j=1}^{n} \left(l_j'(\hat{\theta})\right)^T l_j'(\hat{\theta}) \tag{A22}$$

Below we derive the components of $\widehat{W}$ for an analysis using biomarkers only and an analysis using self-reports and biomarkers combined.

Using biomarkers only

In this analysis, for an individual j in treatment group i in the sub-study we use data from the two biomarker measurements (M_{ij1}, M_{ij2}), which are assumed to have a bivariate normal distribution:

$$\left(M_{ij1}, M_{ij2}\right) \sim BVN\left\{\begin{pmatrix}\mu_{Ti}\\ \mu_{Ti}\end{pmatrix}, \begin{pmatrix}\sigma_{Ti}^2 + \sigma_{Mi}^2 & \sigma_{Ti}^2\\ \sigma_{Ti}^2 & \sigma_{Ti}^2 + \sigma_{Mi}^2\end{pmatrix}\right\} \tag{A23}$$

The contribution to the log likelihood for an individual j in treatment group i is

$$\begin{aligned} l_{ij} = &-\log 2\pi - \frac{1}{2}\log((\sigma_{Mi}^2)^2 + 2\sigma_{Ti}^2\sigma_{Mi}^2) \\ &- \frac{1}{2((\sigma_{Mi}^2)^2 + 2\sigma_{Ti}^2\sigma_{Mi}^2)}\Big\{(\sigma_{Ti}^2 + \sigma_{Mi}^2)\left(M_{ij1} - \mu_{Ti}\right)^2 \\ &+ (\sigma_{Ti}^2 + \sigma_{Mi}^2)\left(M_{ij2} - \mu_{Ti}\right)^2 - 2\sigma_{Ti}^2(M_{ij1} - \mu_{Ti})(M_{ij2} - \mu_{Ti})\Big\} \end{aligned} \tag{A24}$$

We let $\sigma_{Ti}^2 = e^{\theta_{Ti}},\ \sigma_{Qi}^2 = e^{\theta_{Qi}}, \sigma_{Mi}^2 = e^{\theta_{Mi}}$ and $A = (\sigma_{Ti}^2 + \sigma_{Mi}^2)\left(M_{ij1} - \mu_{Ti}\right)^2 + (\sigma_{Ti}^2 + \sigma_{Mi}^2)\left(M_{ij2} - \mu_{Ti}\right)^2 - 2\sigma_{Ti}^2(M_{ij1} - \mu_{Ti})(M_{ij2} - \mu_{Ti})$. The partial derivatives of the log likelihood with respect to each parameter are:

$$\frac{\partial l_{ij}}{\partial \mu_{Ti}} = \frac{(M_{ij1} + M_{ij2} - 2\mu_{Ti})}{e^{\theta_{Mi}} + 2e^{\theta_{Ti}}}$$

$$\frac{\partial l_{ij}}{\partial \theta_{Ti}} = -\frac{e^{\theta_{Ti}}}{(e^{\theta_{Mi}} + 2e^{\theta_{Ti}})} + \frac{e^{\theta_{Ti}}A}{e^{\theta_{Mi}}(e^{\theta_{Mi}} + 2e^{\theta_{Ti}})^2} - \frac{e^{\theta_{Ti}}\left(M_{ij1} - M_{ij2}\right)^2}{2(e^{2\theta_{Mi}} + 2e^{\theta_{Ti}+\theta_{Mi}})}$$

$$\frac{\partial l_{ij}}{\partial \theta_{Mi}} = -\frac{1}{2} - \frac{e^{\theta_{Mi}}}{2(e^{\theta_{Mi}} + 2e^{\theta_{Ti}})} + \frac{(e^{M_i} + e^{\theta_{Ti}})A}{e^{\theta_{Mi}}(e^{\theta_{Mi}} + 2e^{\theta_{Ti}})^2} - \frac{\left(\left(M_{ij1} - \mu_{Ti}\right)^2 + \left(M_{ij2} - \mu_{Ti}\right)^2\right)}{2(e^{\theta_{Mi}} + 2e^{\theta_{Ti}})} \tag{A25}$$

Using self-reports and biomarkers combined

For an individual j in treatment group i who is *not* in the sub-study within which biomarker measurements are made we observe the self-report Q_{ij}. Under the assumption that Q_{ij} is normally distributed with mean $\alpha_{0i} + \alpha_{1i}\mu_{Ti}$ and variance $\alpha_{1i}^2\sigma_{Ti}^2 + \sigma_{Qi}^2$, the contribution to the log likelihood from this individual is

$$l_{ij} = -\frac{1}{2}\log 2\pi - \frac{1}{2}\log\left(\alpha_{1i}^2\sigma_{Ti}^2 + \sigma_{Qi}^2\right) - \frac{1}{2\left(\alpha_{1i}^2\sigma_{Ti}^2 + \sigma_{Qi}^2\right)}\left(Q_{ij} - \alpha_{0i} - \alpha_{1i}\mu_{Ti}\right)^2 \tag{A26}$$

Letting $\sigma_{Ti}^2 = e^{\theta_{Ti}},\ \sigma_{Qi}^2 = e^{\theta_{Qi}}, A = \left(Q_{ij} - \alpha_{0i} - \alpha_{1i}\mu_{Ti}\right)$ and $B = \left(\alpha_{1i}^2e^{\theta_{Ti}} + e^{\theta_{Qi}}\right)$, the partial derivatives of the log likelihood with respect to each parameter are:

$$\frac{\partial l_{ij}}{\partial \mu_{Ti}} = \frac{\alpha_{1i}A}{B}, \frac{\partial l_{ij}}{\partial \alpha_{0i}} = \frac{A}{B}, \frac{\partial l_{ij}}{\partial \alpha_{1i}} = -\frac{\alpha_{1i}e^{\theta_{Ti}}}{B} + \frac{\alpha_{1i}e^{\theta_{Ti}}A^2}{B^2} + \frac{\mu_{Ti}A}{B},$$
$$\frac{\partial l_{ij}}{\partial \theta_{Ti}} = -\frac{\alpha_{1i}^2e^{\theta_{Ti}}}{2B} + \frac{\alpha_{1i}^2e^{\theta_{Ti}}A^2}{2B^2}, \frac{\partial l_{ij}}{\partial \theta_{Qi}} = -\frac{e^{\theta_{Qi}}}{2B} + \frac{e^{\theta_{Qi}}A^2}{2B^2} \tag{A27}$$

For an individual j in treatment group i who *is* in the sub-study within which biomarker measurements are made we observe the self-report Q_{ij} and the two biomarker measurements M_{ij1}, M_{ij2}. It is assumed that these three measurements have a joint normal distribution:

$$\left(M_{ij1}, M_{ij2}, Q_{ij}\right) \sim MVN\left\{\begin{pmatrix}\mu_{Ti}\\ \mu_{Ti}\\ \alpha_{0i}+\alpha_{1i}\mu_{Ti}\end{pmatrix}, \begin{pmatrix}\sigma_{Ti}^2+\sigma_{Mi}^2 & \sigma_{Ti}^2 & \alpha_{1i}\sigma_{Ti}^2\\ \sigma_{Ti}^2 & \sigma_{Ti}^2+\sigma_{Mi}^2 & \alpha_{1i}\sigma_{Ti}^2\\ \alpha_{1i}\sigma_{Ti}^2 & \alpha_{1i}\sigma_{Ti}^2 & \alpha_{1i}^2\sigma_{Ti}^2+\sigma_{Qi}^2\end{pmatrix}\right\} \tag{A28}$$

We let $\sigma_{Ti}^2 = e^{\theta_{Ti}}$, $\sigma_{Qi}^2 = e^{\theta_{Qi}}$, $\sigma_{Mi}^2 = e^{\theta_{Mi}}$. To simplify the notation we also define $C = 1/\{2e^{\theta_{Mi}}(2e^{\theta_{Ti}+\theta_{Qi}} + \alpha_{1i}^2 e^{\theta_{Ti}+\theta_{Mi}} + e^{\theta_{Mi}+\theta_{Qi}})\}$, $D_1 = e^{\theta_{Ti}}\left(e^{\theta_{Qi}} + \alpha_{1i}^2 e^{\theta_{Mi}}\right) + e^{\theta_{Mi}+\theta_{Qi}}$, $D_2 = e^{\theta_{Mi}}(e^{\theta_{Mi}} + 2e^{\theta_{Ti}})$, $D_3 = e^{\theta_{Ti}+\theta_{Qi}}$, $D_4 = \alpha_{1i}e^{\theta_{Ti}+\theta_{Mi}}$, $E_1 = \left(M_{ij1} - \mu_{Ti}\right)^2 + \left(M_{ij2} - \mu_{Ti}\right)^2$, $E_2 = \left(Q_{ij} - \alpha_{0i} - \alpha_{1i}\mu_{Ti}\right)^2$, $E_3 = 2(M_{ij1} - \mu_{Ti})(M_{ij2} - \mu_{Ti})$, $E_4 = 2(Q_{ij} - \alpha_{0i} - \alpha_{1i}\mu_{Ti})(M_{ij1} + M_{ij2} - 2\mu_{Ti})$, $F = (2e^{\theta_{Ti}+\theta_{Qi}} + \alpha_{1i}^2 e^{\theta_{Ti}+\theta_{Mi}} + e^{\theta_{Mi}+\theta_{Qi}})$ and $G = (D_1E_1 + D_2E_2 - D_3E_3 - D_4E_4\ )$. The contribution to the likelihood for individual j in treatment group i who is in the sub-study can be shown to be

$$l_{ij} = -\frac{3}{2}\log 2\pi - \frac{1}{2}\theta_{Mi} - \frac{1}{2}\log F - C \times G \tag{A29}$$

The partial derivatives of the log likelihood with respect to each parameter are:

$$\begin{aligned}
\frac{\partial l_{ij}}{\partial \mu_{Ti}} &= -C\{-2(D_1 - D_3 - D_4\alpha_{1i})\left(M_{ij1} + M_{ij2} - 2\mu_{Ti}\right) - 2(D_2\alpha_{1i} \\
&\quad - 2D_4)\left(Q_{ij} - \alpha_{0i} - \alpha_{1i}\mu_{Ti}\right)\} \\
\frac{\partial l_{ij}}{\partial \alpha_{0i}} &= -C\{-2D_2\left(Q_{ij} - \alpha_{0i} - \alpha_{1i}\mu_{Ti}\right) + 2D_4\left(M_{ij1} + M_{ij2} - 2\mu_{Ti}\right)\} \\
\frac{\partial l_{ij}}{\partial \alpha_{1i}} &= -\frac{\alpha_{i1}e^{\theta_{Ti}+\theta_{Mi}}}{F} + \frac{\alpha_{i1}e^{\theta_{Ti}}G}{F^2} \\
&\quad - C\{e^{\theta_{Ti}+\theta_{Mi}}(2E_1\alpha_{1i} - E_4) - 2D_2\mu_{Ti}\left(Q_{ij} - \alpha_{0i} - \alpha_{1i}\mu_{Ti}\right) \\
&\quad + 2D_4\mu_{Ti}\left(M_{ij1} + M_{ij2} - 2\mu_{Ti}\right)\} \\
\frac{\partial l_{ij}}{\partial \theta_{Ti}} &= -\frac{\left(2e^{\theta_{Ti}+\theta_{Qi}} + \alpha_{1i}^2 e^{\theta_{Ti}+\theta_{Mi}}\right)\left(Fe^{\theta_{Mi}} - G\right)}{2e^{\theta_{Mi}}F^2} \\
&\quad - C\{E_1e^{\theta_{Ti}}\left(e^{\theta_{Qi}} + \alpha_{1i}^2 e^{\theta_{Mi}}\right) + 2E_2e^{\theta_{Ti}+\theta_{Mi}} - E_3D_3 - E_4D_4\}
\end{aligned} \tag{A30}$$

$$\frac{\partial l_{ij}}{\partial \theta_{Qi}} = -\frac{\left(2e^{\theta_{Ti}+\theta_{Qi}} + e^{\theta_{Mi}+\theta_{Qi}}\right)\left(Fe^{\theta_{Mi}} - G\right)}{2e^{\theta_{Mi}}F^2} - C\left\{E_1 e^{Qi}\left(e^{\theta_{Ti}} + e^{\theta_{Mi}}\right) - E_3 D_3\right\}$$

$$\frac{\partial l_{ij}}{\partial \theta_{Mi}} = -\frac{1}{2} - \frac{e^{\theta_{Mi}}\left(\alpha_{1i}^2 e^{\theta_{Ti}} + e^{\theta_{Qi}}\right)}{2F} + \frac{\left(e^{\theta_{Ti}+\theta_{Qi}} + \alpha_{1i}^2 e^{\theta_{Ti}+\theta_{Mi}} + e^{\theta_{Mi}+\theta_{Qi}}\right)G}{e^{\theta_{Mi}}F^2} - C\left\{E_1 e^{\theta_{Mi}}\left(\alpha_{1i}^2 e^{\theta_{Ti}} + e^{\theta_{Qi}}\right) + 2E_2 e^{\theta_{Mi}}\left(e^{\theta_{Mi}} + e^{\theta_{Ti}}\right) - E_4 D_4\right\}$$

The sandwich estimates are incorporated into the R code provided. See Appendix S4 for further details.

Appendix S3: Additional simulation studies to investigate standard error estimates from the MLE analysis when the assumption of multivariate normality is violated

We performed two additional simulation studies, detailed below, to investigate standard error estimates from the MLE analysis when the assumption of multivariate normality is violated, using the standard estimates and using the sandwich estimates.

Additional simulation 1: log normal

The true exposure for an individual j in treatment group i was generated using

$$T_{ij} = \mu_{Ti} \times \epsilon_{Tij}^*$$

where log ϵ_{Tij}^* is generated from a normal distribution with mean 0 and variance σ_{Ti}^2. The kth biomarker measurement for person j in treatment group i was generated using

$$M_{ijk} = T_{ij} \times \epsilon_{Mijk}^*$$

where the log ϵ_{Mijk}^* were generated from a normal distribution with mean 0 and variance σ_{Mi}^2. The self-report measurement for person j in treatment group i was generated using

$$Q_{ij} = (\alpha_{0i} + \alpha_{1i} T_{ij}) \times \epsilon_{Qij}^*$$

where the log ϵ_{Qijk}^* were generated from a normal distribution with mean 0 and variance σ_{Qi}^2. As in the main simulations we used $\mu_{T1} = 4.6$ and $\mu_{T2} = 4.1$. We used $\sigma_{T1}^2 = \sigma_{T2}^2$ =0.005, which was chosen so that the variance of T_{ij} was approximately the same as in the main simulation (0.1). Similarly, we chose $\sigma_{M1}^2 = \sigma_{M2}^2$ =0.009 so that the variance of the biomarker measurements was approximately the same as in the main simulation. The self-

report data were generated under differential error using the same values for α_{0i} and α_{1i} ($i = 1,2$) as in the main simulation ($\alpha_{01} = 0.3, \alpha_{11} = 0.8, \alpha_{02} = 1.5, \alpha_{12} = 0.5$) and we used $\sigma_{Q1}^2 = \sigma_{Q2}^2 = 0.08$, which meant that Q_{1j} and Q_{2j} had approximately the same variances as in the main simulation scenario (a).

Additional simulation 2: mixture of normals

Here, the true exposure for an individual j in treatment group i was generated using

$$T_{ij} = \mu_{Ti} + \epsilon_{Tij}^{**}$$

where for half of the individuals the ϵ_{Tij}^{**} were generated from a normal distribution with mean 0 and variance 0.05 and for the other half the ϵ_{Tij}^{**} were generated from a normal distribution with mean 0 and variance 0.15 (the variances were the same in the two treatment groups); that is, the true intake was generated from a mixture of two normal distributions. The biomarker and self-report measurements were generated as in the main simulation scenario described in section 5.1 of the main text, using $\sigma_{Q1}^2 = \sigma_{Q2}^2 = 0.09$ (scenario (a)).

Additional simulations: results

1000 simulated data sets were generated under each of the additional scenarios outlined above. We performed the same analyses on each data set as in the main scenario. In the analyses using MLE, we estimated the standard errors using both the standard approach and using sandwich estimates.

The results are shown in Tables S3 and S4. The results show that in these realistic departures from multivariate normality the `standard' standard errors obtained from the inverse of the information matrix remain very close to the empirical standard deviations. The sandwich estimates are very close to the standard estimates.

Appendix S4. Example R code

We have provided files containing example R code used in the simulation studies. The files provided are as follows:

- data_simulation_differential_error: generates data as described in the simulation study (Section 5.1) with differential error in the self-report data.
- data_simulation_non-differential_error: generates data as described in the simulation study (Section 5.1) with non-differential error in the self-report data.
- MOM_biomarkers_only: Estimates the intervention effect using the biomarkers only using the method of moments. This gives the estimate $\hat{\theta}_{(\mathrm{i})}$, described in Section 3.1.
- MOM_buonaccorsi_differential_error: Estimates the intervention effect using biomarkers and self-report data combined, using the Buonaccorsi approach described in Section 3.1 under the assumption of differential error in the self-reports (see equations (4)-(6)).
- MOM_buonaccorsi_non-differential_error: Estimates the intervention effect using biomarkers and self-report data combined, using the Buonaccorsi approach described in Section 3.1 under the assumption of non-differential error in the self-reports (see equations (7) and (8)).
- MLE_biomarkers_only: Estimates the intervention effect using the biomarkers only using maximum likelihood. See Section 3.2.
- MLE _differential_error: Estimates the intervention effect using biomarkers and self-report data combined using maximum likelihood, assuming differential error in the self-reports. See Section 3.2.
- MLE _non-differential_error: Estimates the intervention effect using biomarkers and self-report data combined using maximum likelihood, assuming non-differential error in the self-reports. See Section 3.2.
- There are additional files ‘MLE_biomarkers_only_sandwich’, ‘MLE _differential_error_sandwich’ and ‘MLE _non-differential_error_sandwich’ which additionally include the sandwich variance estimates. See Appendix S2.

Table S1 Simulation study results* for data simulated under differential error. Results are shown from three analysis methods all using maximum likelihood estimation: using the biomarker data only, and using both self-reports and biomarkers under the correct assumption of differential error and under the incorrect assumption of non-differential error in the self-reports. Results are shown separately for different values of the error variability in the self-reports ($\sigma_{Q1}^2 = \sigma_{Q2}^2$) and for different proportions of individuals in the calibration sub-study.

(a) $\sigma_{Q1}^2 = \sigma_{Q2}^2 = 0.09$

**Method**	**Bias**	**MSE**	**Emp SD**	**Model SE**	**Cov**	**Eff**
**Calibration sub-study: 10%**						
Biomarkers only	0.002	0.008	0.087	0.089	95.6	11.3
Combined: differential error	0.002	0.005	0.073	0.072	94.3	16.2
Combined: non-differential error [981]	-0.051	0.007	0.063	0.060	83.5	21.6
**Calibration sub-study: 25%**						
Biomarkers only	0.001	0.003	0.056	0.057	94.9	27.4
Combined: differential error	0.000	0.002	0.048	0.048	94.3	37.1
Combined: non-differential error [976]	-0.037	0.003	0.043	0.040	81.6	46.4
**Calibration sub-study: 50%**						
Biomarkers only	-0.000	0.002	0.041	0.040	94.0	50.4
Combined: differential error	-0.000	0.001	0.037	0.036	94.1	62.2
Combined: non-differential error [983]	-0.032	0.002	0.033	0.032	80.6	76.6
**Calibration sub-study: 100%**						
Biomarkers only	-0.000	0.001	0.029	0.028	94.4	100.0
Combined: differential error	0.000	0.001	0.029	0.028	94.6	99.7
Combined: non-differential error [993]	-0.028	0.001	0.026	0.025	79.0	122.4

(b) $\sigma_{Q1}^2 = \sigma_{Q2}^2 = 0.3$

**Method**	**Bias**	**MSE**	**Emp SD**	**Model SE**	**Cov**	**Eff**
**Calibration sub-study: 10%**						
Biomarkers only	0.002	0.008	0.087	0.089	95.6	11.3
Combined: differential error	0.002	0.007	0.083	0.083	95.4	12.4
Combined: non-differential error [990]**	-0.024	0.006	0.074	0.074	93.4	15.5
**Calibration sub-study: 25%**						
Biomarkers only	0.001	0.003	0.056	0.057	94.9	27.4
Combined: differential error	0.000	0.003	0.054	0.053	94.4	29.9
Combined: non-differential error [993]**	-0.023	0.003	0.049	0.048	91.8	36.3
**Calibration sub-study: 50%**						
Biomarkers only	-0.000	0.002	0.041	0.040	94.0	50.4
Combined: differential error	-0.000	0.002	0.040	0.038	93.9	53.9
Combined: non-differential error [998]**	-0.022	0.002	0.037	0.035	89.6	63.3
**Calibration sub-study: 100%**						
Biomarkers only	-0.000	0.001	0.029	0.028	94.4	100.0
Combined: differential error	-0.000	0.001	0.029	0.028	94.7	100.3
Combined: non-differential error [997]**	-0.017	0.001	0.028	0.027	88.6	113.5

(c) $\sigma_{Q1}^2 = \sigma_{Q2}^2 = 0.5$

Method	Bias	MSE	Emp SD	Model SE	Cov	Eff
**Calibration sub-study: 10%**						
Biomarkers only	0.002	0.008	0.087	0.089	95.6	11.3
Combined: differential error [999]*	0.002	0.007	0.086	0.087	95.9	11.6
Combined: non-differential error [995]**	-0.018	0.006	0.078	0.078	94.9	14.0
**Calibration sub-study: 25%**						
Biomarkers only	0.001	0.003	0.056	0.057	94.9	27.4
Combined: differential error	0.000	0.003	0.055	0.055	94.3	28.6
Combined: non-differential error [992]**	-0.018	0.003	0.050	0.050	93.4	33.8
**Calibration sub-study: 50%**						
Biomarkers only	-0.000	0.002	0.041	0.040	94.0	50.4
Combined: differential error	-0.000	0.002	0.041	0.039	93.6	51.8
Combined: non-differential error [996]**	-0.016	0.002	0.038	0.036	91.7	59.4
**Calibration sub-study: 100%**						
Biomarkers only	-0.000	0.001	0.029	0.028	94.4	100.0
Combined: differential error	-0.000	0.001	0.029	0.028	94.5	100.1
Combined: non-differential error [999]**	-0.010	0.001	0.028	0.027	92.3	108.8

(d) $\sigma_{Q1}^2 = \sigma_{Q2}^2 = 0.7$

Method	Bias	MSE	Emp SD	Model SE	Cov	Eff
**Calibration sub-study: 10%**						
Biomarkers only	0.002	0.008	0.087	0.089	95.6	11.3
Combined: differential error [999]*	0.002	0.008	0.087	0.088	95.6	11.2
Combined: non-differential error [991]**	-0.012	0.007	0.081	0.081	95.3	13.1
**Calibration sub-study: 25%**						
Biomarkers only	0.001	0.003	0.056	0.057	94.9	27.4
Combined: differential error	0.000	0.003	0.056	0.056	94.3	27.7
Combined: non-differential error [996]**	-0.012	0.003	0.052	0.052	94.6	32.1
**Calibration sub-study: 50%**						
Biomarkers only	-0.000	0.002	0.041	0.040	94.0	50.4
Combined: differential error	-0.000	0.002	0.041	0.040	93.7	51.1
Combined: non-differential error [999]**	-0.010	0.002	0.039	0.038	93.0	56.4
**Calibration sub-study: 100%**						
Biomarkers only	-0.000	0.001	0.029	0.028	94.4	100.0
Combined: differential error	-0.000	0.001	0.029	0.028	94.5	100.0
Combined: non-differential error [999]**	-0.006	0.001	0.029	0.028	93.6	105.4

*'Bias': Average bias in the intervention effect estimate across 1000 simulations.
'MSE': mean squared error of the intervention effect estimate across 1000 simulations.
'Emp SD': standard deviation of the 1000 intervention effect estimates.
'Model SE': Square-root of the mean of the variances of the 1000 intervention effect estimates.
'Cov': Percentage of the 1000 95% confidence intervals for the intervention effect which contained the true value.
'Eff': Ratio of the variance of the 1000 intervention effect estimates obtained when the biomarkers are available in 100% of individuals to the variance of the 1000 intervention effect estimates from a given method, expressed as a percentage

** We were unable to obtain variance estimates in a small number of simulations. In these situations the number of simulations on which the results are based is given in square brackets.

Table S2 Simulation study results* for data simulated under non-differential error. Results are shown from three analysis methods all using maximum likelihood estimation: using the biomarker data only, and using both self-reports and biomarkers under the assumptions of differential error and non-differential error in the self-reports. Results are shown separately for different values of the error variability in the self-reports ($\sigma_{Q1}^2 = \sigma_{Q2}^2$) and for different proportions of individuals in the calibration sub-study.

(i) $\sigma_{Q1}^2 = \sigma_{Q2}^2 = 0.09$

Method	Bias	MSE	Emp SD	Model SE	Cov	Eff
**Calibration sub-study: 10%**						
Biomarkers only	0.002	0.008	0.087	0.089	95.6	11.3
Combined: differential error	0.001	0.005	0.071	0.072	95.4	17.0
Combined: non-differential error [989] **	-0.012	0.003	0.056	0.062	94.1	27.4
**Calibration sub-study: 25%**						
Biomarkers only	0.001	0.003	0.056	0.057	94.9	27.4
Combined: differential error	-0.000	0.002	0.048	0.047	94.8	37.8
Combined : non-differential error [996]**	-0.002	0.002	0.041	0.042	94.3	50.0
**Calibration sub-study: 50%**						
Biomarkers only	-0.000	0.002	0.041	0.040	94.0	50.4
Combined: differential error	-0.000	0.001	0.037	0.036	93.6	62.5
Combined: non-differential error	-0.001	0.001	0.034	0.032	94.1	74.9
**Calibration sub-study: 100%**						
Biomarkers only	-0.000	0.001	0.029	0.028	94.4	100.0
Combined: differential error	-0.000	0.001	0.029	0.028	94.3	99.7
Combined: non-differential error	-0.000	0.001	0.028	0.027	94.1	112.2

(ii) $\sigma_{Q1}^2 = \sigma_{Q2}^2 = 0.3$

Method	Bias	MSE	Emp SD	Model SE	Cov	Eff
**Calibration sub-study: 10%**						
Biomarkers only	0.002	0.008	0.087	0.089	95.6	11.3
Combined: differential error	0.002	0.007	0.083	0.083	95.5	12.4
Combined: non-differential error [997]**	0.002	0.005	0.072	0.073	94.2	16.6
**Calibration sub-study: 25%**						
Biomarkers only	0.001	0.003	0.056	0.057	94.9	27.4
Combined: differential error	0.000	0.003	0.053	0.053	94.2	30.2
Combined: non-differential error	0.001	0.002	0.048	0.048	95.0	36.9
**Calibration sub-study: 50%**						
Biomarkers only	-0.000	0.002	0.041	0.040	94.0	50.4
Combined: differential error	-0.000	0.002	0.040	0.038	93.8	53.8
Combined: non-differential error	-0.000	0.001	0.037	0.036	94.0	61.8
**Calibration sub-study: 100%**						
Biomarkers only	-0.000	0.001	0.029	0.028	94.4	100.0
Combined: differential error	-0.000	0.001	0.029	0.028	94.5	99.9
Combined: non-differential error	-0.000	0.001	0.028	0.027	94.9	107.1

(iii) $\sigma_{Q1}^2 = \sigma_{Q2}^2 = 0.5$

Method	Bias	MSE	Emp SD	Model SE	Cov	Eff
**Calibration sub-study: 10%**						
Biomarkers only	0.002	0.008	0.087	0.089	95.6	11.3
Combined: differential error	0.002	0.007	0.086	0.087	95.8	11.6
Combined: non-differential error [998]**	0.003	0.006	0.080	0.080	94.8	13.5
**Calibration sub-study: 25%**						
Biomarkers only	0.001	0.003	0.056	0.057	94.9	27.4
Combined: differential error	0.000	0.003	0.055	0.055	94.5	28.3
Combined: non-differential error [999]**	0.001	0.003	0.052	0.052	95.1	31.9
**Calibration sub-study: 50%**						
Biomarkers only	-0.000	0.002	0.041	0.040	94.0	50.4
Combined: differential error	-0.000	0.002	0.041	0.039	94.0	52.1
Combined: non-differential error [999]**	-0.000	0.002	0.039	0.038	94.0	55.8
**Calibration sub-study: 100%**						
Biomarkers only	-0.000	0.001	0.029	0.028	94.4	100.0
Combined: differential error	-0.000	0.001	0.029	0.028	94.6	99.9
Combined: non-differential error	-0.000	0.001	0.029	0.028	94.8	103.9

(iv) $\sigma_{Q1}^2 = \sigma_{Q2}^2 = 0.7$

Method	Bias	MSE	Emp SD	Model SE	Cov	Eff
**Calibration sub-study: 10%**						
Biomarkers only	0.002	0.008	0.087	0.089	95.6	11.3
Combined: differential error	0.002	0.008	0.088	0.088	95.3	11.2
Combined: non-differential error [997]**	0.003	0.007	0.083	0.084	94.7	12.4
**Calibration sub-study: 25%**						
Biomarkers only	0.001	0.003	0.056	0.057	94.9	27.4
Combined: differential error	0.000	0.003	0.056	0.056	95.1	27.7
Combined: non-differential error	0.001	0.003	0.054	0.055	94.5	29.9
**Calibration sub-study: 50%**						
Biomarkers only	-0.000	0.002	0.041	0.040	94.0	50.4
Combined: differential error	-0.000	0.002	0.041	0.040	93.5	51.2
Combined: non-differential error [999]**	-0.000	0.002	0.040	0.039	94.2	53.3
**Calibration sub-study: 100%**						
Biomarkers only	-0.000	0.001	0.029	0.028	94.4	100.0
Combined: differential error	-0.000	0.001	0.029	0.028	94.4	100.0
Combined: non-differential error	-0.000	0.001	0.029	0.028	94.9	102.5

*'Bias': Average bias in the intervention effect estimate across 1000 simulations.
'MSE': mean squared error of the intervention effect estimate across 1000 simulations.
'Emp SD': standard deviation of the 1000 intervention effect estimates.
'Model SE': Square-root of the mean of the variances of the 1000 intervention effect estimates.
'Cov': Percentage of the 1000 95% confidence intervals for the intervention effect which contained the true value.
'Eff': Ratio of the variance of the 1000 intervention effect estimates obtained when the biomarkers are available in 100% of individuals to the variance of the 1000 intervention effect estimates from a given method, expressed as a percentage

** We were unable to obtain variance estimates in a small number of simulations. In these situations the number of simulations on which the results are based is given in square brackets.

Table S3 Simulation study results* from additional simulation scenario 1 (lognormal)

(i) Using method of moments

**Method**	**Bias**	**MSE**	**Emp SD**	**Model SE**	**Cov**	**Eff**
**Calibration sub-study: 10%**	-0.002	0.007	0.084	0.086	96.1	11.2
Biomarkers only	-0.002	0.007	0.082	0.082	95.0	11.8
Combined: differential error [994]	-0.021	0.006	0.074	0.075	94.3	14.4
Combined: non-differential error						
**Calibration sub-study: 25%**						
Biomarkers only	-0.003	0.003	0.054	0.054	95.1	27.5
Combined: differential error	-0.004	0.003	0.051	0.052	95.0	29.8
Combined: non-differential error	-0.023	0.003	0.047	0.049	93.1	35.6
**Calibration sub-study: 50%**						
Biomarkers only	-0.004	0.002	0.039	0.038	94.2	50.8
Combined: differential error	-0.004	0.001	0.038	0.037	94.2	54.4
Combined: non-differential error	-0.022	0.002	0.036	0.036	90.5	61.9
**Calibration sub-study: 100%**						
Biomarkers only	-0.004	0.001	0.028	0.027	93.9	100.0
Combined: differential error						
Combined: non-differential error	-0.017	0.001	0.027	0.026	90.4	111.4

(ii) Using MLE

**Method**	**Bias**	**MSE**	**Emp SD**	**Model SE: standard estimates**	**Model SE: sandwich estimates**	**Cov****	**Eff**
**Calibration sub-study: 10%**							
Biomarkers only	-0.002	0.007	0.084	0.085	0.085	95.9	11.2
Combined: differential error	-0.001	0.006	0.079	0.079	0.080	94.9	12.6
Combined: non-differential error [991]	-0.017	0.006	0.076	0.071	0.074[1]	92.4	13.7
**Calibration sub-study: 25%**							
Biomarkers only	-0.003	0.003	0.054	0.055	0.055	95.0	27.5
Combined: differential error	-0.003	0.003	0.051	0.051	0.051	94.7	30.1
Combined: non-differential error [992]	-0.025	0.003	0.047	0.047	0.050[2]	91.1	35.9
**Calibration sub-study: 50%**							
Biomarkers only	-0.004	0.002	0.039	0.039	0.039	94.0	50.7
Combined: differential error	-0.004	0.001	0.038	0.037	0.037	94.3	54.3
Combined: non-differential error [995]	-0.024	0.002	0.035	0.034	0.035[1]	87.6	63.9
**Calibration sub-study: 100%**							
Biomarkers only	-0.004	0.001	0.028	0.027	0.027	93.9	100.0
Combined: differential error	-0.004	0.001	0.028	0.027	0.027	93.8	99.9
Combined: non-differential error	-0.019	0.001	0.026	0.026	0.026[2]	87.3	113.6

*'Bias': Average bias in the intervention effect estimate across 1000 simulations.
'MSE': mean squared error of the intervention effect estimate across 1000 simulations.
'Emp SD': standard deviation of the 1000 intervention effect estimates.
'Model SE': Square-root of the mean of the variances of the 1000 intervention effect estimates.
'Cov': Percentage of the 1000 95% confidence intervals for the intervention effect which contained the true value.
'Eff': Ratio of the variance of the 1000 intervention effect estimates obtained when the biomarkers are available in 100% of individuals to the variance of the 1000 intervention effect estimates from a given method, expressed as a percentage

**coverage is based on model SEs obtained using the standard approach.
[1] 1 outlier omitted. [2] 3 outliers omitted. [3]

Table S4 Simulation study results from additional simulation scenario 2 (mixture of normals).

(i) Using method of moments

Method	Bias	MSE	Emp SD	Model SE	Cov	Eff
**Calibration sub-study: 10%**	0.002	0.008	0.088	0.089	95.3	11.0
Biomarkers only	0.001	0.005	0.074	0.074	94.7	15.5
Combined: differential error [997]	-0.034	0.007	0.075	0.073	92.4	
Combined: non-differential error						
**Calibration sub-study: 25%**						
Biomarkers only	0.001	0.003	0.056	0.057	94.8	27.1
Combined: differential error	-0.000	0.002	0.048	0.048	94.5	37.1
Combined: non-differential error	-0.033	0.003	0.048	0.048	88.7	36.9
**Calibration sub-study: 50%**						
Biomarkers only	0.000	0.002	0.041	0.040	94.3	50.3
Combined: differential error	-0.000	0.001	0.037	0.036	93.8	62.8
Combined: non-differential error	-0.031	0.002	0.037	0.036	84.7	63.5
**Calibration sub-study: 100%**						
Biomarkers only	-0.000	0.001	0.029	0.028	95.1	100.0
Combined: differential error	-	-	-	-	-	-
Combined: non-differential error [993]	-0.023	0.001	0.027	0.027	85.9	115.1

(ii) Using MLE

Method	Bias	MSE	Emp SD	Model SE: standard estimates	Model SE: sandwich estimates	Cov*	Eff
**Calibration sub-study: 10%**							
Biomarkers only	0.002	0.008	0.088	0.089	0.089	95.2	11.0
Combined: differential error	0.001	0.005	0.073	0.072	0.071	95.2	16.2
Combined: non-differential error [980]	-0.051	0.007	0.063	0.059	0.059[1]	83.1	21.6
**Calibration sub-study: 25%**							
Biomarkers only	0.001	0.003	0.056	0.057	0.057	94.9	27.1
Combined: differential error	0.001	0.002	0.048	0.048	0.047	94.2	37.2
Combined: non-differential error [970]	-0.038	0.003	0.043	0.040	0.043[2]	82.5	46.9
**Calibration sub-study: 50%**							
Biomarkers only	0.000	0.002	0.041	0.040	0.040	94.4	50.3
Combined: differential error	0.000	0.001	0.037	0.036	0.036	93.8	62.4
Combined: non-differential error [981]	-0.032	0.002	0.033	0.030	0.035	80.1	77.9
**Calibration sub-study: 100%**							
Biomarkers only	-0.000	0.001	0.029	0.028	0.028	95.1	100.0
Combined: differential error	-0.000	0.001	0.029	0.028	0.028	95.0	99.0
Combined: non-differential error [987]	-0.028	0.001	0.026	0.025	0.028[3]	79.1	122.7

*'Bias': Average bias in the intervention effect estimate across 1000 simulations.
'MSE': mean squared error of the intervention effect estimate across 1000 simulations.
'Emp SD': standard deviation of the 1000 intervention effect estimates.
'Model SE': Square-root of the mean of the variances of the 1000 intervention effect estimates.
'Cov': Percentage of the 1000 95% confidence intervals for the intervention effect which contained the true value.
'Eff': Ratio of the variance of the 1000 intervention effect estimates obtained when the biomarkers are available in 100% of individuals to the variance of the 1000 intervention effect estimates from a given method, expressed as a percentage

*coverage is based on model SEs obtained using the standard approach.
[1] 2 outliers omitted. [2] 5 outliers omitted. [3] 1 outlier omitted.
