# Supplementary material for: Statistical issues related to dietary intake as the response variable in intervention trials
Source: Stat Med. 2016 Jun 20;35(25):4493–508. doi: 10.1002/sim.7011 (PMC5050089; doi:10.1002/sim.7011)
Supplement: Supplementary file 3 — Supporting info item [file SIM-35-4493-s003.docx]

#-------------------------------------------

#Generates data as described in the simulation study (Section 5.1) with #non-differential error in the self-report data.

#-------------------------------------------

#packages required

library(mnormt)

#data generation parameters

mu.t1<-4.6

mu.t2<-4.1

sigsq.t1<-0.1

sigsq.t2<-0.1

sigsq.m1<-0.2

sigsq.m2<-0.2

alpha0.1<-0.9

alpha1.1<-0.65

alpha0.2<-0.9

alpha1.2<-0.65

#number in trial and treatment groups

n<-1000

n1<-n/2

n2<-n/2

group<-c(rep(1,n1),rep(2,n2))

#true intake

t1<-rnorm(n1,mu.t1,sqrt(sigsq.t1))

t2<-rnorm(n2,mu.t2,sqrt(sigsq.t2))

t<-c(t1,t2)

#biomarker measurements (.i denotes the first repeat and .ii denotes the second repeat)

m1.i<-t1+rnorm(n1,0,sqrt(sigsq.m1))

m1.ii<-t1+rnorm(n1,0,sqrt(sigsq.m1))

m2.i<-t2+rnorm(n2,0,sqrt(sigsq.m2))

m2.ii<-t2+rnorm(n2,0,sqrt(sigsq.m2))

m.i<-c(m1.i,m2.i)

m.ii<-c(m1.ii,m2.ii)

#self-reports

q1<-alpha0.1+alpha1.1*t1+rnorm(n1,0,sigsq.q1)

q2<-alpha0.2+alpha1.2*t2+rnorm(n2,0,sigsq.q2)

q<-c(q1,q2)

#generate missing data in the biomarkers

val<-rbinom(n,1,val.pc) #indicator of being in the validation sub-study

val1<-val[1:n1]

val2<-val[(n1+1):n]

m.i<-ifelse(val==1,m.i,NA)

m.ii<-ifelse(val==1,m.ii,NA)

m1.i<-ifelse(val1==1,m1.i,NA)

m1.ii<-ifelse(val1==1,m1.ii,NA)

m2.i<-ifelse(val2==1,m2.i,NA)

m2.ii<-ifelse(val2==1,m2.ii,NA)

ns<-sum(1-is.na(m.i)) #number in substudy

n1s<-sum(1-is.na(m.i[group==1])) #number in substudy in treatment group 1

n2s<-sum(1-is.na(m.i[group==2])) #number in substudy in treatment group 2

m1.bar<-(m1.i+m1.ii)/2

m2.bar<-(m2.i+m2.ii)/2

m.bar<-(m.i+m.ii)/2
