# Supplementary material for: Statistical issues related to dietary intake as the response variable in intervention trials
Source: Stat Med. 2016 Jun 20;35(25):4493–508. doi: 10.1002/sim.7011 (PMC5050089; doi:10.1002/sim.7011)
Supplement: Supplementary file 4 — Supporting info item [file SIM-35-4493-s004.docx]

#---------------------------------------------

#Estimates the intervention effect using the biomarkers only using the #method of moments.

#This gives the estimate theta_(i), described in Section 3.1.

#---------------------------------------------

#intervention effect estimate

theta.i<-mean(m2.bar[val2==1])-mean(m1.bar[val1==1])

#estimating equations

psi.1<-(m1.bar-mean(m1.bar[val1==1]))

psi.2<-(m2.bar-mean(m2.bar[val2==1]))

A1.hat<-1

B1.hat<-sum(psi.1^2,na.rm=T)/n1s

var.matrix.1<-(1/n1s)*B1.hat*((1/A1.hat)^2)

A2.hat<-1

B2.hat<-sum(psi.2^2,na.rm=T)/n2s

var.matrix.2<-(1/n2s)*B2.hat*((1/A2.hat)^2)

#variance of intervention effect estimate

var.theta.i<-var.matrix.1+var.matrix.2
