# Supplementary material for: Statistical issues related to dietary intake as the response variable in intervention trials
Source: Stat Med. 2016 Jun 20;35(25):4493–508. doi: 10.1002/sim.7011 (PMC5050089; doi:10.1002/sim.7011)
Supplement: Supplementary file 5 — Supporting info item [file SIM-35-4493-s005.docx]

#---------------------------------------------

#Estimates the intervention effect using biomarkers and self-report data #combined, using the Buonaccorsi approach described in Section 3.1 under #the assumption #of differential error in the self-reports (see equations #(4)-(6) and Appendix 1).

#This is the estimate theta_c under differential error in the self-reports

#---------------------------------------------

#estimate the alpha parameters

alpha.11.est<-cov(q1[val1==1],m1.bar[val1==1])/cov(m1.i[val1==1],m1.ii[val1==1])

alpha.01.est<-mean(q1)-alpha.11.est*mean(m1.bar[val1==1])

alpha.12.est<-cov(q2[val2==1],m2.bar[val2==1])/cov(m2.i[val2==1],m2.ii[val2==1])

alpha.02.est<-mean(q2)-alpha.12.est*mean(m2.bar[val2==1])

#other estimates required

theta.q1<-mean(q1)

theta.q2<-mean(q2)

theta.q1.s<-mean(q1[val1==1])

theta.q2.s<-mean(q2[val2==1])

theta.m1<-mean(m1.bar[val1==1])

theta.m2<-mean(m2.bar[val2==1])

#two estimates of treatment effect, to be combined below

theta.1<-mean(m2.bar[val2==1])-mean(m1.bar[val1==1])

theta.2.a<-(mean(q2)-mean(q2[val2==1]))/alpha.12.est

theta.2.b<-(mean(q1)-mean(q1[val1==1]))/alpha.11.est

theta.2<-theta.2.a-theta.2.b+theta.1

#variance-covariance matrix for full set of parameters (order: alpha0i, alpha1i, mu.qi, mu.mi, separately by treatment group i) - using estimating equations

A1.hat<-(1/n)*matrix(c(-n,-sum(m1.i[val1==1])*n/n1s,0,0,0,

-sum(m1.ii[val1==1])*n/n1s,-sum(m1.i[val1==1]*m1.ii[val1==1])*n/n1s,0,0,0,

0,0,-n,0,0,

0,0,0,-n,0,

0,0,0,0,-n),nrow=5,ncol=5,byrow=T)

A2.hat<-(1/n)*matrix(c(-n,-sum(m2.i[val2==1])*n/n2s,0,0,0,

-sum(m2.ii[val2==1])*n/n2s,-sum(m2.i[val2==1]*m2.ii[val2==1])*n/n2s,0,0,0,

0,0,-n,0,0,

0,0,0,-n,0,

0,0,0,0,-n),nrow=5,ncol=5,byrow=T)

m1.bar.alt<-ifelse(is.na(m1.bar)==T,0,m1.bar)

m1.i.alt<-ifelse(is.na(m1.i)==T,0,m1.i)

m1.ii.alt<-ifelse(is.na(m1.ii)==T,0,m1.ii)

B1.hat<-matrix(0,nrow=5,ncol=5)

for(k in 1:n1){

psi1.a<-c((q1-alpha.01.est-alpha.11.est*m1.i.alt)[k]*val1[k]*(n)/(n1s),

(q1-alpha.01.est-alpha.11.est*m1.i.alt)[k]*m1.ii.alt[k]*val1[k]*(n)/(n1s),

(q1-theta.q1)[k]*n/n1,

(q1-theta.q1.s)[k]*val1[k]*(n)/(n1s),

(m1.bar.alt-theta.m1)[k]*val1[k]*(n)/(n1s))

psi1.b<-psi1.a%*%t(psi1.a)

B1.hat<-B1.hat+psi1.b

}

B1.hat<-B1.hat/n

m2.bar.alt<-ifelse(is.na(m2.bar)==T,0,m2.bar)

m2.i.alt<-ifelse(is.na(m2.i)==T,0,m2.i)

m2.ii.alt<-ifelse(is.na(m2.ii)==T,0,m2.ii)

B2.hat<-matrix(0,nrow=5,ncol=5)

for(k in 1:n2){

psi2.a<-c((q2-alpha.02.est-alpha.12.est*m2.i.alt)[k]*val2[k]*(n)/(n2s),

(q2-alpha.02.est-alpha.12.est*m2.i.alt)[k]*m2.ii.alt[k]*val2[k]*(n)/(n2s),

(q2-theta.q2)[k]*n/n2,

(q2-theta.q2.s)[k]*val2[k]*(n)/(n2s),

(m2.bar.alt-theta.m2)[k]*val2[k]*(n)/(n2s))

psi2.b<-psi2.a%*%t(psi2.a)

B2.hat<-B2.hat+psi2.b

}

B2.hat<-B2.hat/n

var.matrix.1<-(1/n)*solve(A1.hat)%*%B1.hat%*%t(solve(A1.hat))

var.matrix.2<-(1/n)*solve(A2.hat)%*%B2.hat%*%t(solve(A2.hat))

#use the above results to find variance-covariance matrix for theta.A1.1 and theta.A1.2

#where var.theta.m.MOM is the variance of the biomarkers-only estimate of the intervention effect

theta.2.a.var<-(var.matrix.2[3,3]+var.matrix.2[4,4]-2*var.matrix.2[3,4])/(alpha.12.est^2)+

var.matrix.2[2,2]*(((theta.q2-theta.q2.s)/(alpha.12.est^2))^2)-

2*(var.matrix.2[2,3]-var.matrix.2[2,4])*((theta.q2-theta.q2.s)/(alpha.12.est^3))

theta.2.b.var<-(var.matrix.1[3,3]+var.matrix.1[4,4]-2*var.matrix.1[3,4])/(alpha.11.est^2)+

var.matrix.1[2,2]*(((theta.q1-theta.q1.s)/(alpha.11.est^2))^2)-

2*(var.matrix.1[2,3]-var.matrix.1[2,4])*((theta.q1-theta.q1.s)/(alpha.11.est^3))

theta.2.a.bio.cov<-var.matrix.2[3,5]/alpha.12.est-

var.matrix.2[4,5]/alpha.12.est-

var.matrix.2[2,5]*((theta.q2-theta.q2.s)/alpha.12.est)

theta.2.b.bio.cov<-var.matrix.1[3,5]/alpha.11.est-

var.matrix.1[4,5]/alpha.11.est-

var.matrix.1[2,5]*((theta.q1-theta.q1.s)/alpha.11.est)

theta.2.var<-theta.2.a.var+theta.2.b.var+var.theta.m.MOM+2*theta.2.a.bio.cov+2*theta.2.b.bio.cov

theta.12.cov<-var.theta.m.MOM+theta.2.a.bio.cov+theta.2.b.bio.cov

theta.var<-matrix(c(var.theta.m.MOM,theta.12.cov,theta.12.cov,theta.2.var),nrow=2,ncol=2)

#combine the two estimates

theta.buon.vec<-matrix(c(theta.1,theta.2),nrow=2,ncol=1)

ones.vector<-matrix(c(1,1),nrow=2,ncol=1)

#intervention effect estimate

theta.c<-solve(t(ones.vector)%*%solve(theta.var)%*%ones.vector)%*%t(ones.vector)%*%solve(theta.var)%*%theta.buon.vec

#variance of intervention effect estimate

var.theta.c<-solve(t(ones.vector)%*%solve(theta.var)%*%ones.vector)
