# Supplementary material for: Statistical issues related to dietary intake as the response variable in intervention trials
Source: Stat Med. 2016 Jun 20;35(25):4493–508. doi: 10.1002/sim.7011 (PMC5050089; doi:10.1002/sim.7011)
Supplement: Supplementary file 6 — Supporting info item [file SIM-35-4493-s006.docx]

#---------------------------------------------

#Estimates the intervention effect using biomarkers and self-report data #combined, using the Buonaccorsi approach described in Section 3.1 under #the assumption of non-differential error in the self-reports (see #equations (7) and (8)).

#This is the estimate theta_c under non-differential error in the self-#reports

#---------------------------------------------

#estimate the alpha parameters

alpha.1.est<-cov(q[val==1],m.bar[val==1])/cov(m.i[val==1],m.ii[val==1])

alpha.0.est<-mean(q)-alpha.1.est*mean(m.bar[val==1])

#other estimates required

theta.q1<-mean(q1)

theta.q2<-mean(q2)

theta.m1<-mean(m1.bar[val1==1])

theta.m2<-mean(m2.bar[val2==1])

#two estimates of treatment effect, to be combined below

theta.1<-theta.m2-theta.m1

theta.2<-(theta.q2-theta.q1)/alpha.1.est

#variance-covariance matrix for full set of parameters (order: alpha0, alpha1, mu.q1, mu.q2, mu.m1, mu.m2) - using estimating equations

A.hat<-(1/n)*matrix(c(-n,-sum(m.i[val==1])*n/ns,0,0,0,0,

-sum(m.ii[val==1])*n/ns,-sum(m.i[val==1]*m.ii[val==1])*n/ns,0,0,0,0,

0,0,-n,0,0,0,

0,0,0,-n,0,0,

0,0,0,0,-n,0,

0,0,0,0,0,-n),nrow=6,ncol=6,byrow=T)

m.i.alt<-ifelse(is.na(m.i)==T,0,m.i)

m.ii.alt<-ifelse(is.na(m.ii)==T,0,m.ii)

m.bar.alt<-ifelse(is.na(m.bar)==T,0,m.bar)

group1<-ifelse(group==1,1,0)

group2<-ifelse(group==2,1,0)

B.hat<-matrix(0,nrow=6,ncol=6)

for(k in 1:n){

psi.a<-c((q-alpha.0.est-alpha.1.est*m.i.alt)[k]*val[k]*(n)/(ns),

(q-alpha.0.est-alpha.1.est*m.i.alt)[k]*m.ii.alt[k]*val[k]*(n)/(ns),

(q-theta.q1)[k]*group1[k]*n/n1,

(q-theta.q2)[k]*group2[k]*n/n2,

(m.bar.alt-theta.m1)[k]*val[k]*group1[k]*(n)/(n1s),

(m.bar.alt-theta.m2)[k]*val[k]*group2[k]*(n)/(n2s))

psi.b<-psi.a%*%t(psi.a)

B.hat<-B.hat+psi.b

}

B.hat<-B.hat/n

var.matrix<-(1/n)*solve(A.hat)%*%B.hat%*%t(solve(A.hat))

#use the above results to find variance-covariance matrix for theta.A3.1 and theta.A3.2

#where var.theta.m.MOM is the variance of the biomarkers-only estimate of the intervention effect

theta.2.var<-(var.matrix[3,3]/(alpha.1.est^2))+var.matrix[2,2]*((theta.q1/(alpha.1.est^2))^2)-2*var.matrix[2,3]*(theta.q1/(alpha.1.est^3))+

(var.matrix[4,4]/(alpha.1.est^2))+var.matrix[2,2]*((theta.q2/(alpha.1.est^2))^2)-2*var.matrix[2,4]*(theta.q2/(alpha.1.est^3))-

2*(var.matrix[3,4]/(alpha.1.est^2)+var.matrix[2,2]*theta.q1*theta.q2/(alpha.1.est^4)-

var.matrix[2,4]*theta.q1/(alpha.1.est^3)-var.matrix[2,3]*theta.q2/(alpha.1.est^3))

theta.12.cov<-var.matrix[3,5]/alpha.1.est+var.matrix[4,6]/alpha.1.est-

var.matrix[3,6]/alpha.1.est-var.matrix[4,5]/alpha.1.est-

var.matrix[2,5]*theta.q1/(alpha.1.est^2)-var.matrix[2,6]*theta.q2/(alpha.1.est^2)+

var.matrix[2,5]*theta.q2/(alpha.1.est^2)+var.matrix[2,6]*theta.q1/(alpha.1.est^2)

theta.var<-matrix(c(var.theta.m.MOM,theta.12.cov,theta.12.cov,theta.2.var),nrow=2,ncol=2)

#combine the two estimates

theta.buon.vec<-matrix(c(theta.1,theta.2),nrow=2,ncol=1)

ones.vector<-matrix(c(1,1),nrow=2,ncol=1)

#intervention effect estimate

theta.c<-solve(t(ones.vector)%*%solve(theta.var)%*%ones.vector)%*%t(ones.vector)%*%solve(theta.var)%*%theta.buon.vec

#variance of intervention effect estimate

var.theta.c<-solve(t(ones.vector)%*%solve(theta.var)%*%ones.vector)
