# Supplementary material for: Statistical issues related to dietary intake as the response variable in intervention trials
Source: Stat Med. 2016 Jun 20;35(25):4493–508. doi: 10.1002/sim.7011 (PMC5050089; doi:10.1002/sim.7011)
Supplement: Supplementary file 7 — Supporting info item [file SIM-35-4493-s007.docx]

#---------------------------------------------

#Estimates the intervention effect using the biomarkers only using #maximum likelihood. See Section 3.2.

#---------------------------------------------

#arranging the data for use in the log likelihood function

data.matrix.validation.monly.1<-as.matrix(cbind(m1.i[val1==1],m1.ii[val1==1]))

data.matrix.validation.monly.2<-as.matrix(cbind(m2.i[val2==1],m2.ii[val2==1]))

#log likelihood function

lik.method<-function(params){

mu1<-params[1]

mu2<-params[2]

logsigmasq.t<-params[3]

logsigmasq.m<-params[4]

sigmasq.t<-exp(logsigmasq.t)

sigmasq.m<-exp(logsigmasq.m)

mean.m1.i<-mu1

mean.m1.ii<-mu1

mean.m2.i<-mu2

mean.m2.ii<-mu2

var.m1.i<-sigmasq.t+sigmasq.m

var.m1.ii<-sigmasq.t+sigmasq.m

var.m2.i<-sigmasq.t+sigmasq.m

var.m2.ii<-sigmasq.t+sigmasq.m

cov.m1.i.ii<-sigmasq.t

cov.m2.i.ii<-sigmasq.t

mean.vector.validation.1<-c(mean.m1.i,mean.m1.ii)

mean.vector.validation.2<-c(mean.m2.i,mean.m2.ii)

var.matrix.validation.1<-matrix(c(var.m1.i,cov.m1.i.ii,cov.m1.i.ii,var.m1.ii),nrow=2,ncol=2)

var.matrix.validation.2<-matrix(c(var.m2.i,cov.m2.i.ii,cov.m2.i.ii,var.m2.ii),nrow=2,ncol=2)

loglik.validation.1<--sum(dmnorm(data.matrix.validation.monly.1,mean.vector.validation.1,

var.matrix.validation.1,log=TRUE))

loglik.validation.2<--sum(dmnorm(data.matrix.validation.monly.2,mean.vector.validation.2,

var.matrix.validation.2,log=TRUE))

loglik.total<-loglik.validation.1+loglik.validation.2

loglik.total

}

#maximising the likelihood

start.values<-c(mu.t1,mu.t2,log(sigsq.t1),log(sigsq.m1))

loglik.fit<-optim(start.values, lik.method,method ="L-BFGS-B",lower = -Inf, upper = Inf,hessian = TRUE)

#estimating parameter variances

varcov.matrix<-solve(fdHess(loglik.fit$par,lik.method)$Hessian)

#intervention effect estimate

theta<-loglik.fit$par[2]-loglik.fit$par[1]

#variance of intervention effect estimate

var.theta<-diag(varcov.matrix)[2]+diag(varcov.matrix)[1]-2*varcov.matrix[1,2]
