# Supplementary material for: Statistical issues related to dietary intake as the response variable in intervention trials
Source: Stat Med. 2016 Jun 20;35(25):4493–508. doi: 10.1002/sim.7011 (PMC5050089; doi:10.1002/sim.7011)
Supplement: Supplementary file 9 — Supporting info item [file SIM-35-4493-s009.docx]

#---------------------------------------------

#Estimates the intervention effect using biomarkers and self-report data #combined using maximum likelihood,

#assuming non-differential error in the self-reports. See Section 3.2.

#---------------------------------------------

#arranging the data for use in the log likelihood function

data.matrix.validation.1<-as.matrix(cbind(q1[val1==1],m1.i[val1==1],m1.ii[val1==1]))

data.matrix.validation.2<-as.matrix(cbind(q2[val2==1],m2.i[val2==1],m2.ii[val2==1]))

data.matrix.nonvalidation.1<-q1[val1==0]

data.matrix.nonvalidation.2<-q2[val2==0]

#log likelihood function

lik.method<-function(params){

mu1<-params[1]

mu2<-params[2]

alpha0<-params[3]

alpha1<-params[4]

logsigmasq.t<-params[5]

logsigmasq.q<-params[6]

logsigmasq.m<-params[7]

sigmasq.t<-exp(logsigmasq.t)

sigmasq.q<-exp(logsigmasq.q)

sigmasq.m<-exp(logsigmasq.m)

mean.q1<-alpha0+alpha1*mu1

mean.q2<-alpha0+alpha1*mu2

mean.m1.i<-mu1

mean.m1.ii<-mu1

mean.m2.i<-mu2

mean.m2.ii<-mu2

var.q1<-(alpha1^2)*sigmasq.t+sigmasq.q

var.q2<-(alpha1^2)*sigmasq.t+sigmasq.q

var.m1.i<-sigmasq.t+sigmasq.m

var.m1.ii<-sigmasq.t+sigmasq.m

var.m2.i<-sigmasq.t+sigmasq.m

var.m2.ii<-sigmasq.t+sigmasq.m

cov.qm1.i<-alpha1*sigmasq.t

cov.qm1.ii<-alpha1*sigmasq.t

cov.qm2.i<-alpha1*sigmasq.t

cov.qm2.ii<-alpha1*sigmasq.t

cov.m1.i.ii<-sigmasq.t

cov.m2.i.ii<-sigmasq.t

mean.vector.validation.1<-c(mean.q1,mean.m1.i,mean.m1.ii)

mean.vector.validation.2<-c(mean.q2,mean.m2.i,mean.m2.ii)

mean.vector.nonvalidation.1<-mean.q1

mean.vector.nonvalidation.2<-mean.q2

var.matrix.validation.1<-matrix(c(var.q1,cov.qm1.i,cov.qm1.ii,

cov.qm1.i,var.m1.i,cov.m1.i.ii,

cov.qm1.ii,cov.m1.i.ii,var.m1.ii),nrow=3,ncol=3)

var.matrix.validation.2<-matrix(c(var.q2,cov.qm2.i,cov.qm2.ii,

cov.qm2.i,var.m2.i,cov.m2.i.ii,

cov.qm2.ii,cov.m2.i.ii,var.m2.ii),nrow=3,ncol=3)

var.matrix.nonvalidation.1<-var.q1

var.matrix.nonvalidation.2<-var.q2

loglik.validation.1<--sum(dmnorm(data.matrix.validation.1,mean.vector.validation.1,var.matrix.validation.1,log=TRUE))

loglik.validation.2<--sum(dmnorm(data.matrix.validation.2,mean.vector.validation.2,var.matrix.validation.2,log=TRUE))

loglik.nonvalidation.1<--sum(dmnorm(data.matrix.nonvalidation.1,mean.vector.nonvalidation.1,var.matrix.nonvalidation.1,log=TRUE))

loglik.nonvalidation.2<--sum(dmnorm(data.matrix.nonvalidation.2,mean.vector.nonvalidation.2,var.matrix.nonvalidation.2,log=TRUE))

loglik.total<-loglik.validation.1+loglik.validation.2+loglik.nonvalidation.1+loglik.nonvalidation.2

loglik.total

}

#maximising the likelihood

start.values<-c(mu.t1,mu.t2,alpha0.1,alpha1.1,log(sigsq.t1),log(sigsq.q1),log(sigsq.m1))

loglik.fit<-optim(start.values, lik.method,method ="L-BFGS-B",lower = -Inf, upper = Inf,hessian = TRUE)

#estimating parameter variances

varcov.matrix<-solve(fdHess(loglik.fit$par,lik.method)$Hessian)

#intervention effect estimate

theta<-loglik.fit$par[2]-loglik.fit$par[1]

#variance of intervenion effect estimate

var.theta<-diag(varcov.matrix)[2]+diag(varcov.matrix)[1]-2*varcov.matrix[1,2]
