# Supplementary material for: Statistical issues related to dietary intake as the response variable in intervention trials
Source: Stat Med. 2016 Jun 20;35(25):4493–508. doi: 10.1002/sim.7011 (PMC5050089; doi:10.1002/sim.7011)
Supplement: Supplementary file 10 — Supporting info item [file SIM-35-4493-s010.docx]

#---------------------------------------------

#Estimates the intervention effect using the biomarkers only using #maximum likelihood. See Section 3.2.

#Includes sandwich variance estimates (see Appendix 2).

#---------------------------------------------

#arranging the data for use in the log likelihood function

data.matrix.validation.monly.1<-as.matrix(cbind(m1.i[val1==1],m1.ii[val1==1]))

data.matrix.validation.monly.2<-as.matrix(cbind(m2.i[val2==1],m2.ii[val2==1]))

#log likelihood function

lik.method<-function(params){

mu1<-params[1]

mu2<-params[2]

logsigmasq.t<-params[3]

logsigmasq.m<-params[4]

sigmasq.t<-exp(logsigmasq.t)

sigmasq.m<-exp(logsigmasq.m)

mean.m1.i<-mu1

mean.m1.ii<-mu1

mean.m2.i<-mu2

mean.m2.ii<-mu2

var.m1.i<-sigmasq.t+sigmasq.m

var.m1.ii<-sigmasq.t+sigmasq.m

var.m2.i<-sigmasq.t+sigmasq.m

var.m2.ii<-sigmasq.t+sigmasq.m

cov.m1.i.ii<-sigmasq.t

cov.m2.i.ii<-sigmasq.t

mean.vector.validation.1<-c(mean.m1.i,mean.m1.ii)

mean.vector.validation.2<-c(mean.m2.i,mean.m2.ii)

var.matrix.validation.1<-matrix(c(var.m1.i,cov.m1.i.ii,cov.m1.i.ii,var.m1.ii),nrow=2,ncol=2)

var.matrix.validation.2<-matrix(c(var.m2.i,cov.m2.i.ii,cov.m2.i.ii,var.m2.ii),nrow=2,ncol=2)

loglik.validation.1<--sum(dmnorm(data.matrix.validation.monly.1,mean.vector.validation.1,

var.matrix.validation.1,log=TRUE))

loglik.validation.2<--sum(dmnorm(data.matrix.validation.monly.2,mean.vector.validation.2,

var.matrix.validation.2,log=TRUE))

loglik.total<-loglik.validation.1+loglik.validation.2

loglik.total

}

#maximising the likelihood

start.values<-c(mu.t1,mu.t2,log(sigsq.t1),log(sigsq.m1))

loglik.fit<-optim(start.values, lik.method,method ="L-BFGS-B",lower = -Inf, upper = Inf,hessian = TRUE)

#Inverse of information matrix

varcov.matrix<-solve(fdHess(loglik.fit$par,lik.method)$Hessian)

#intervention effect estimate

theta.m.MLE<-loglik.fit$par[2]-loglik.fit$par[1]

#--------------------------------------------

#sandwich estimates for parameter variances

#[Note that up to this point this is the same as the file MLE_biomarkers_only]

#--------------------------------------------

#extracting parameter estimates obtained using maximum likelihood above

mu1<-loglik.fit$par[1]

mu2<-loglik.fit$par[2]

logsigmasq.t<-loglik.fit$par[3]

logsigmasq.m<-loglik.fit$par[4]

sigmasq.t<-exp(logsigmasq.t)

sigmasq.m<-exp(logsigmasq.m)

#individual contributions to the derivative of the loglik - validation study- treament group 1

A<-((m.i[val==1]-mu1)^2)*(sigmasq.t+sigmasq.m)+((m.ii[val==1]-mu1)^2)*(sigmasq.t+sigmasq.m)-2*sigmasq.t*(m.i[val==1]-mu1)*(m.ii[val==1]-mu1)

score.val.grp1.mu1<-(m.i[val==1]+m.ii[val==1]-2*mu1)/(sigmasq.m+2*sigmasq.t)

score.val.grp1.logsigmaq.t<--sigmasq.t/(sigmasq.m^+2*sigmasq.t)+

(sigmasq.t/(sigmasq.m*((sigmasq.m+2*sigmasq.t)^2)))*A-

(sigmasq.t/(2*((sigmasq.m^2)+2*sigmasq.t*sigmasq.m)))*((m.i[val==1]-m.ii[val==1])^2)

score.val.grp1.logsigmaq.m<--0.5-0.5*sigmasq.m/(sigmasq.m+2*sigmasq.t)+

((sigmasq.m+sigmasq.t)/(sigmasq.m*((sigmasq.m+2*sigmasq.t)^2)))*A-

(1/(2*(sigmasq.m+2*sigmasq.t)))*((m.i[val==1]-mu1)^2+(m.ii[val==1]-mu1)^2)

#individual contributions to the derivative of the loglik - validation study - treament group 2

score.val.grp2.mu2<-(m.i[val==1]+m.ii[val==1]-2*mu2)/(sigmasq.m+2*sigmasq.t)

score.val.grp2.logsigmaq.t<--sigmasq.t/(sigmasq.m^+2*sigmasq.t)+

(sigmasq.t/(sigmasq.m*((sigmasq.m+2*sigmasq.t)^2)))*A-

(sigmasq.t/(2*((sigmasq.m^2)+2*sigmasq.t*sigmasq.m)))*((m.i[val==1]-m.ii[val==1])^2)

score.val.grp2.logsigmaq.m<--0.5-0.5*sigmasq.m/(sigmasq.m+2*sigmasq.t)+

((sigmasq.m+sigmasq.t)/(sigmasq.m*((sigmasq.m+2*sigmasq.t)^2)))*A-

(1/(2*(sigmasq.m+2*sigmasq.t)))*((m.i[val==1]-mu2)^2+(m.ii[val==1]-mu2)^2)

#individual contributions to the derivative of the loglik - combined across the two treatment groups

if.func<-function(x){ifelse(is.na(x)==T,0,x)}

score.mu1<-if.func((group[val==1]==1)*score.val.grp1.mu1)

score.mu2<-if.func((group[val==1]==2)*score.val.grp2.mu2)

score.logsigmasq.t<-if.func((group[val==1]==1)*score.val.grp1.logsigmaq.t)+if.func((group[val==1]==2)*score.val.grp2.logsigmaq.t)

score.logsigmasq.m<-if.func((group[val==1]==1)*score.val.grp1.logsigmaq.m)+if.func((group[val==1]==2)*score.val.grp2.logsigmaq.m)

#calculating the sandwich estimate

scorevar<-matrix(0,nrow=4,ncol=4)

for(k in 1:length(m.i[val==1])){

temp<-as.vector(c(score.mu1[k],score.mu2[k],score.logsigmasq.t[k],score.logsigmasq.m[k]))

scorevar<-scorevar+temp%*%t(temp)

}

sandwich.est<-varcov.matrix%*%scorevar%*%varcov.matrix

#variance of intervention effect estimate, using the sandwich estimate of the variance

var.theta.m.MLE.sandwich<-diag(sandwich.est)[2]+diag(sandwich.est)[1]-2*sandwich.est[1,2]
