# Supplementary material for: Statistical issues related to dietary intake as the response variable in intervention trials
Source: Stat Med. 2016 Jun 20;35(25):4493–508. doi: 10.1002/sim.7011 (PMC5050089; doi:10.1002/sim.7011)
Supplement: Supplementary file 11 — Supporting info item [file SIM-35-4493-s011.docx]

#---------------------------------------------

#Estimates the intervention effect using biomarkers and self-report data #combined using maximum likelihood,

#assuming differential error in the self-reports. See Section 3.2.

#Includes sandwich variance estimates (see Appendix 2).

#---------------------------------------------

#arranging the data for use in the log likelihood function

data.matrix.validation.1<-as.matrix(cbind(q1[val1==1],m1.i[val1==1],m1.ii[val1==1]))

data.matrix.validation.2<-as.matrix(cbind(q2[val2==1],m2.i[val2==1],m2.ii[val2==1]))

data.matrix.nonvalidation.1<-q1[val1==0]

data.matrix.nonvalidation.2<-q2[val2==0]

#log likelihood function

lik.method<-function(params){

mu1<-params[1]

mu2<-params[2]

alpha01<-params[3]

alpha11<-params[4]

alpha02<-params[5]

alpha12<-params[6]

logsigmasq.t<-params[7]

logsigmasq.q<-params[8]

logsigmasq.m<-params[9]

sigmasq.t<-exp(logsigmasq.t)

sigmasq.q<-exp(logsigmasq.q)

sigmasq.m<-exp(logsigmasq.m)

mean.q1<-alpha01+alpha11*mu1

mean.q2<-alpha02+alpha12*mu2

mean.m1.i<-mu1

mean.m1.ii<-mu1

mean.m2.i<-mu2

mean.m2.ii<-mu2

var.q1<-(alpha11^2)*sigmasq.t+sigmasq.q

var.q2<-(alpha12^2)*sigmasq.t+sigmasq.q

var.m1.i<-sigmasq.t+sigmasq.m

var.m1.ii<-sigmasq.t+sigmasq.m

var.m2.i<-sigmasq.t+sigmasq.m

var.m2.ii<-sigmasq.t+sigmasq.m

cov.qm1.i<-alpha11*sigmasq.t

cov.qm1.ii<-alpha11*sigmasq.t

cov.qm2.i<-alpha12*sigmasq.t

cov.qm2.ii<-alpha12*sigmasq.t

cov.m1.i.ii<-sigmasq.t

cov.m2.i.ii<-sigmasq.t

mean.vector.validation.1<-c(mean.q1,mean.m1.i,mean.m1.ii)

mean.vector.validation.2<-c(mean.q2,mean.m2.i,mean.m2.ii)

mean.vector.nonvalidation.1<-mean.q1

mean.vector.nonvalidation.2<-mean.q2

var.matrix.validation.1<-matrix(c(var.q1,cov.qm1.i,cov.qm1.ii,

cov.qm1.i,var.m1.i,cov.m1.i.ii,

cov.qm1.ii,cov.m1.i.ii,var.m1.ii),nrow=3,ncol=3)

var.matrix.validation.2<-matrix(c(var.q2,cov.qm2.i,cov.qm2.ii,

cov.qm2.i,var.m2.i,cov.m2.i.ii,

cov.qm2.ii,cov.m2.i.ii,var.m2.ii),nrow=3,ncol=3)

var.matrix.nonvalidation.1<-var.q1

var.matrix.nonvalidation.2<-var.q2

loglik.validation.1<--sum(dmnorm(data.matrix.validation.1,mean.vector.validation.1,var.matrix.validation.1,log=TRUE))

loglik.validation.2<--sum(dmnorm(data.matrix.validation.2,mean.vector.validation.2,var.matrix.validation.2,log=TRUE))

loglik.nonvalidation.1<--sum(dmnorm(data.matrix.nonvalidation.1,mean.vector.nonvalidation.1,var.matrix.nonvalidation.1,log=TRUE))

loglik.nonvalidation.2<--sum(dmnorm(data.matrix.nonvalidation.2,mean.vector.nonvalidation.2,var.matrix.nonvalidation.2,log=TRUE))

loglik.total<-loglik.validation.1+loglik.validation.2+loglik.nonvalidation.1+loglik.nonvalidation.2

loglik.total

}

#maximising the likelihood

start.values<-c(mu.t1,mu.t2,alpha0.1,alpha1.1,alpha0.2,alpha1.2,log(sigsq.t1),log(sigsq.q1),log(sigsq.m1))

loglik.fit<-optim(start.values, lik.method,method ="L-BFGS-B",lower = -Inf, upper = Inf,hessian = TRUE)

#Inverse of information matrix

varcov.matrix<-solve(fdHess(loglik.fit$par,lik.method)$Hessian)

#intervention effect estimate

theta.MLE<-loglik.fit$par[2]-loglik.fit$par[1]

#--------------------------------------------

#sandwich estimates for parameter variances

#[Note that up to this point this is the same as the file MLE_biomarkers_only]

#--------------------------------------------

#extracting parameter estimates obtained using maximum likelihood above

mu1<-loglik.fit$par[1]

mu2<-loglik.fit$par[2]

alpha01<-loglik.fit$par[3]

alpha11<-loglik.fit$par[4]

alpha02<-loglik.fit$par[5]

alpha12<-loglik.fit$par[6]

logsigmasq.t<-loglik.fit$par[7]

logsigmasq.q<-loglik.fit$par[8]

logsigmasq.m<-loglik.fit$par[9]

sigmasq.t<-exp(logsigmasq.t)

sigmasq.q<-exp(logsigmasq.q)

sigmasq.m<-exp(logsigmasq.m)

#individual contributions to the derivative of the score functions for individuals not in the validation study: treatment group 1

A<-(q-alpha01-alpha11*mu1)

B<-(alpha11^2)*sigmasq.t+sigmasq.q

score.nonval.grp1.mu1<-alpha11*A/B

score.nonval.grp1.alpha01<-A/B

score.nonval.grp1.alpha11<--alpha11*sigmasq.t/B+alpha11*sigmasq.t*(A^2)/(B^2)+mu1*A/B

score.nonval.grp1.logsigmasq.t<--0.5*(alpha11^2)*sigmasq.t/B+0.5*(alpha11^2)*sigmasq.t*(A^2)/(B^2)

score.nonval.grp1.logsigmasq.q<--0.5*sigmasq.q/B+0.5*sigmasq.q*(A^2)/(B^2)

#individual contributions to the derivative of the score functions for individuals not in the validation study: treatment group 2

A<-(q-alpha02-alpha12*mu2)

B<-(alpha12^2)*sigmasq.t+sigmasq.q

score.nonval.grp2.mu2<-alpha12*A/B

score.nonval.grp2.alpha02<-A/B

score.nonval.grp2.alpha12<--alpha12*sigmasq.t/B+alpha12*sigmasq.t*(A^2)/(B^2)+mu2*A/B

score.nonval.grp2.logsigmasq.t<--0.5*(alpha12^2)*sigmasq.t/B+0.5*(alpha12^2)*sigmasq.t*(A^2)/(B^2)

score.nonval.grp2.logsigmasq.q<--0.5*sigmasq.q/B+0.5*sigmasq.q*(A^2)/(B^2)

#individual contributions to the derivative of the loglik for individuals in the validation study: treatment group 1

F<-2*sigmasq.t*sigmasq.q+(alpha11^2)*sigmasq.t*sigmasq.m+sigmasq.m*sigmasq.q

C<-1/(2*sigmasq.m*F)

D1<-sigmasq.t*(sigmasq.q+(alpha11^2)*sigmasq.m)+sigmasq.m*sigmasq.q

D2<-sigmasq.m*(sigmasq.m+2*sigmasq.t)

D3<-sigmasq.t*sigmasq.q

D4<-alpha11*sigmasq.t*sigmasq.m

E1<-(m.i-mu1)^2+(m.ii-mu1)^2

E2<-(q-alpha01-alpha11*mu1)^2

E3<-2*(m.i-mu1)*(m.ii-mu1)

E4<-2*(q-alpha01-alpha11*mu1)*(m.i+m.ii-2*mu1)

G<-E1*D1+E2*D2-E3*D3-E4*D4

score.val.grp1.mu1<--C*(-2*D1*(m.i+m.ii-2*mu1)-2*D2*alpha11*(q-alpha01-alpha11*mu1)+

2*D3*(m.i+m.ii-2*mu1)+2*D4*alpha11*(m.i+m.ii-2*mu1)+4*D4*(q-alpha01-alpha11*mu1))

score.val.grp1.alpha01<--C*(-2*D2*(q-alpha01-alpha11*mu1)+2*D4*(m.i+m.ii-2*mu1))

score.val.grp1.alpha11<--alpha11*sigmasq.t*sigmasq.m/F+alpha11*sigmasq.t*G/(F^2)-

C*(2*E1*alpha11*sigmasq.t*sigmasq.m-2*mu1*D2*(q-alpha01-alpha11*mu1)-E4*sigmasq.t*sigmasq.m+2*D4*mu1*(m.i+m.ii-2*mu1))

score.val.grp1.logsigmasq.t<--0.5*(2*sigmasq.t*sigmasq.q+(alpha11^2)*sigmasq.t*sigmasq.m)/F+

0.5*(1/sigmasq.m)*(2*sigmasq.t*sigmasq.q+(alpha11^2)*sigmasq.t*sigmasq.m)*G/(F^2)-

C*(E1*sigmasq.t*(sigmasq.q+(alpha11^2)*sigmasq.m)+2*E2*sigmasq.t*sigmasq.m-E3*D3-E4*D4)

score.val.grp1.logsigmasq.q<--0.5*(2*sigmasq.t*sigmasq.q+sigmasq.m*sigmasq.q)/F+

0.5*(1/sigmasq.m)*(2*sigmasq.t*sigmasq.q+sigmasq.m*sigmasq.q)*G/(F^2)-

C*(E1*sigmasq.q*(sigmasq.t+sigmasq.m)-E3*D3)

score.val.grp1.logsigmasq.m<--0.5-0.5*((alpha11^2)*sigmasq.t*sigmasq.m+sigmasq.m*sigmasq.q)/F+

(1/sigmasq.m)*(sigmasq.t*sigmasq.q+(alpha11^2)*sigmasq.t*sigmasq.m+sigmasq.m*sigmasq.q)*G/(F^2)-

C*(E1*sigmasq.m*((alpha11^2)*sigmasq.t+sigmasq.q)+2*E2*sigmasq.m*(sigmasq.m+sigmasq.t)-E4*D4)

#individual contributions to the derivative of the score functions for individuals in the validation study: treatment group 2

F<-2*sigmasq.t*sigmasq.q+(alpha12^2)*sigmasq.t*sigmasq.m+sigmasq.m*sigmasq.q

C<-1/(2*sigmasq.m*F)

D1<-sigmasq.t*(sigmasq.q+(alpha12^2)*sigmasq.m)+sigmasq.m*sigmasq.q

D2<-sigmasq.m*(sigmasq.m+2*sigmasq.t)

D3<-sigmasq.t*sigmasq.q

D4<-alpha12*sigmasq.t*sigmasq.m

E1<-(m.i-mu2)^2+(m.ii-mu2)^2

E2<-(q-alpha02-alpha12*mu2)^2

E3<-2*(m.i-mu2)*(m.ii-mu2)

E4<-2*(q-alpha02-alpha12*mu2)*(m.i+m.ii-2*mu2)

G<-E1*D1+E2*D2-E3*D3-E4*D4

score.val.grp2.mu2<--C*(-2*D1*(m.i+m.ii-2*mu2)-2*D2*alpha12*(q-alpha02-alpha12*mu2)+

2*D3*(m.i+m.ii-2*mu2)+2*D4*alpha12*(m.i+m.ii-2*mu2)+4*D4*(q-alpha02-alpha12*mu2))

score.val.grp2.alpha02<--C*(-2*D2*(q-alpha02-alpha12*mu2)+2*D4*(m.i+m.ii-2*mu2))

score.val.grp2.alpha12<--alpha12*sigmasq.t*sigmasq.m/F+alpha12*sigmasq.t*G/(F^2)-

C*(2*E1*alpha12*sigmasq.t*sigmasq.m-2*mu2*D2*(q-alpha02-alpha12*mu2)-E4*sigmasq.t*sigmasq.m+2*D4*mu2*(m.i+m.ii-2*mu2))

score.val.grp2.logsigmasq.t<--0.5*(2*sigmasq.t*sigmasq.q+(alpha12^2)*sigmasq.t*sigmasq.m)/F+

0.5*(1/sigmasq.m)*(2*sigmasq.t*sigmasq.q+(alpha12^2)*sigmasq.t*sigmasq.m)*G/(F^2)-

C*(E1*sigmasq.t*(sigmasq.q+(alpha12^2)*sigmasq.m)+2*E2*sigmasq.t*sigmasq.m-E3*D3-E4*D4)

score.val.grp2.logsigmasq.q<--0.5*(2*sigmasq.t*sigmasq.q+sigmasq.m*sigmasq.q)/F+

0.5*(1/sigmasq.m)*(2*sigmasq.t*sigmasq.q+sigmasq.m*sigmasq.q)*G/(F^2)-

C*(E1*sigmasq.q*(sigmasq.t+sigmasq.m)-E3*D3)

score.val.grp2.logsigmasq.m<--0.5-0.5*((alpha12^2)*sigmasq.t*sigmasq.m+sigmasq.m*sigmasq.q)/F+

(1/sigmasq.m)*(sigmasq.t*sigmasq.q+(alpha12^2)*sigmasq.t*sigmasq.m+sigmasq.m*sigmasq.q)*G/(F^2)-

C*(E1*sigmasq.m*((alpha12^2)*sigmasq.t+sigmasq.q)+2*E2*sigmasq.m*(sigmasq.m+sigmasq.t)-E4*D4)

#individual contributions to the derivative of the score functions - combined across the two treatment groups

if.func<-function(x){ifelse(is.na(x)==T,0,x)}

score.mu1<-if.func((val==1 & group==1)*score.val.grp1.mu1)+if.func((val==0 & group==1)*score.nonval.grp1.mu1)

score.mu2<-if.func((val==1 & group==2)*score.val.grp2.mu2)+if.func((val==0 & group==2)*score.nonval.grp2.mu2)

score.alpha01<-if.func((val==1 & group==1)*score.val.grp1.alpha01)+if.func((val==0 & group==1)*score.nonval.grp1.alpha01)

score.alpha02<-if.func((val==1 & group==2)*score.val.grp2.alpha02)+if.func((val==0 & group==2)*score.nonval.grp2.alpha02)

score.alpha11<-if.func((val==1 & group==1)*score.val.grp1.alpha11)+if.func((val==0 & group==1)*score.nonval.grp1.alpha11)

score.alpha12<-if.func((val==1 & group==2)*score.val.grp2.alpha12)+if.func((val==0 & group==2)*score.nonval.grp2.alpha12)

score.logsigmasq.t<-if.func((val==1 & group==1)*score.val.grp1.logsigmasq.t)+if.func((val==0 & group==1)*score.nonval.grp1.logsigmasq.t)+

if.func((val==1 & group==2)*score.val.grp2.logsigmasq.t)+if.func((val==0 & group==2)*score.nonval.grp2.logsigmasq.t)

score.logsigmasq.q<-if.func((val==1 & group==1)*score.val.grp1.logsigmasq.q)+if.func((val==0 & group==1)*score.nonval.grp1.logsigmasq.q)+

if.func((val==1 & group==2)*score.val.grp2.logsigmasq.q)+if.func((val==0 & group==2)*score.nonval.grp2.logsigmasq.q)

score.logsigmasq.m<-if.func((val==1 & group==1)*score.val.grp1.logsigmasq.m)+if.func((val==1 & group==2)*score.val.grp2.logsigmasq.m)

#calculating the sandwich estimate

scorevar<-matrix(0,nrow=9,ncol=9)

for(k in 1:n){

temp<-as.vector(c(score.mu1[k],score.mu2[k],score.alpha01[k],score.alpha11[k],score.alpha02[k],score.alpha12[k],

score.logsigmasq.t[k],score.logsigmasq.q[k],score.logsigmasq.m[k]))

scorevar<-scorevar+temp%*%t(temp)

}

sandwich.est<-varcov.matrix%*%scorevar%*%varcov.matrix

#variance of intervention effect estimate, using the sandwich estimate of the variance

var.theta.sandwich<-diag(sandwich.est)[2]+diag(sandwich.est)[1]-2*sandwich.est[1,2]
